# Population structure of an Antarctic aquatic cyanobacterium

**DOI:** 10.1186/s40168-022-01404-x

**Published:** 2022-12-02

**Authors:** Pratibha Panwar, Timothy J. Williams, Michelle A. Allen, Ricardo Cavicchioli

**Affiliations:** School of Biotechnology and Biomolecular Sciences, UNSW Sydney, Sydney, New South Wales 2052 Australia

**Keywords:** Antarctic microbiology, Cyanobacteria, *Synechococcus*, *Regnicoccus*, Nit1C, AsnB, Metagenome-assembled genomes, Phylotype, Ecotype, Population structure, Niche adaptation, Host-virus interactions, Specialist virus, Generalist virus, Meromictic lake, Microbial food web

## Abstract

**Background:**

Ace Lake is a marine-derived, stratified lake in the Vestfold Hills of East Antarctica with an upper oxic and lower anoxic zone. Cyanobacteria are known to reside throughout the water column. A *Synechococcus*-like species becomes the most abundant member in the upper sunlit waters during summer while persisting annually even in the absence of sunlight and at depth in the anoxic zone. Here, we analysed ~ 300 Gb of Ace Lake metagenome data including 59 *Synechococcus*-like metagenome-assembled genomes (MAGs) to determine depth-related variation in cyanobacterial population structure. Metagenome data were also analysed to investigate viruses associated with this cyanobacterium and the host’s capacity to defend against or evade viruses.

**Results:**

A single *Synechococcus*-like species was found to exist in Ace Lake, *Candidatus* Regnicoccus frigidus sp. nov., consisting of one phylotype more abundant in the oxic zone and a second phylotype prevalent in the oxic-anoxic interface and surrounding depths. An important aspect of genomic variation pertained to nitrogen utilisation, with the capacity to perform cyanide assimilation and asparagine synthesis reflecting the depth distribution of available sources of nitrogen. Both specialist (host specific) and generalist (broad host range) viruses were identified with a predicted ability to infect *Ca.* Regnicoccus frigidus. Host-virus interactions were characterised by a depth-dependent distribution of virus type (e.g. highest abundance of specialist viruses in the oxic zone) and host phylotype capacity to defend against (e.g. restriction-modification, retron and BREX systems) and evade viruses (cell surface proteins and cell wall biosynthesis and modification enzymes).

**Conclusion:**

In Ace Lake, specific environmental factors such as the seasonal availability of sunlight affects microbial abundances and the associated processes that the microbial community performs. Here, we find that the population structure for *Ca.* Regnicoccus frigidus has evolved differently to the other dominant phototroph in the lake, *Candidatus* Chlorobium antarcticum. The geography (i.e. Antarctica), limnology (e.g. stratification) and abiotic (e.g. sunlight) and biotic (e.g. microbial interactions) factors determine the types of niches that develop in the lake. While the lake community has become increasingly well studied, metagenome-based studies are revealing that niche adaptation can take many paths; these paths need to be determined in order to make reasonable predictions about the consequences of future ecosystem perturbations.

Video Abstract

**Supplementary Information:**

The online version contains supplementary material available at 10.1186/s40168-022-01404-x.

## Background

The *Synechococcus* genus consists of unicellular cyanobacteria that are abundant in the euphotic zone of aquatic environments. Together with *Prochlorococcus*, these cyanobacteria are the most abundant photoautotrophs in marine environments and contribute significantly to global primary production [1–3].

Characterising *Synechococcus* species has proven difficult because many have very similar morphology despite possessing distinct GC content, and the polyphyly of organisms classified as *Synechococcus* has been well noted [4, 5]. Molecular markers successfully used to characterise the phylogeny of *Synechococcus* species include the 16S–23S rRNA internally transcribed spacer region and the DNA-directed RNA polymerase (*rpoC1*), nitrate reductase (*narB*), nitrogen regulator (*ntcA*), phycoerythrin (*cpeB*) and cytochrome b6 (*petB*) genes [6–12]. To date, more than 20 marine *Synechococcus* clades have been identified using these markers [11–15]. A recent study using the GTDB (Genome Taxonomy Database) approach [16] with additional phylogenomic analysis has reassigned 1085 members of the *Synechococcus* genus (also referred to as the ‘*Synechococcus* collective’) to 15 genera within five distinct orders: (i) Synechococcales (composed of nine genera including *Synechococcus* genus and a monophyletic group of three genera *Regnicoccus*, *Cyanobium* and *Vulcanococcus*), (ii) Cyanobacteriales (one genus), (iii) Leptococcales (two genera), (iv) Thermosynechococcales (two genera) and (v) Neosynechococcales (one genus) [5]. *Synechococcus* ecotypes in marine environments have been determined to be influenced by environmental factors (e.g. iron concentration and sunlight availability), and genetic variation has been linked to the capacity to utilise sunlight (including chlorophyll a concentration), metabolise nitrogen and adapt to specific temperatures and salinity [13, 15, 17, 18].

**Fig. 1:**
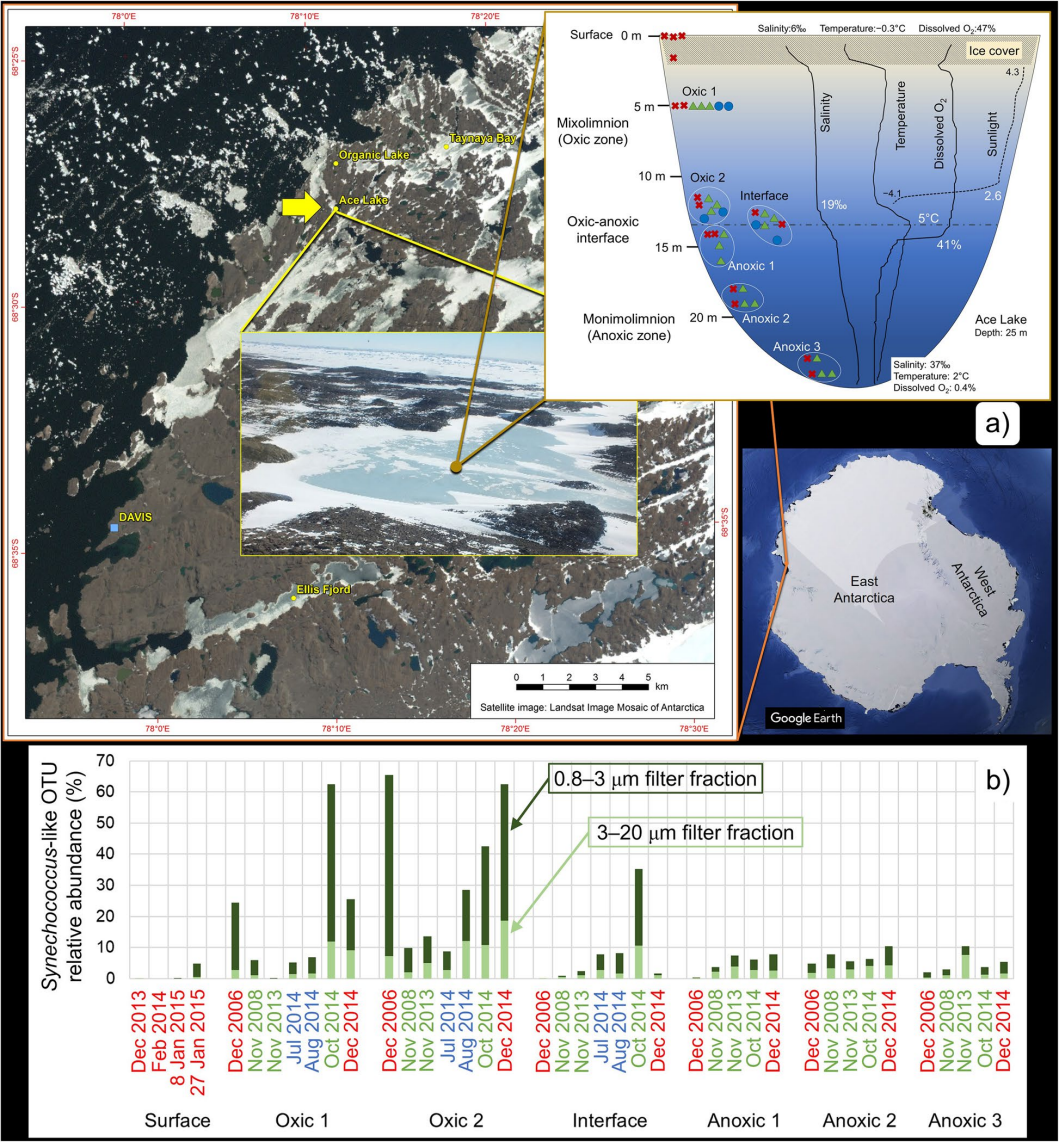
Ace Lake in Antarctica and the abundance of *Synechococcus*-like species. **a** Images showing location of Ace Lake (68° 28′ S, 78° 11′ E) in the Vestfold Hills in East Antarctica. Depiction of Ace Lake meromictic strata: oxic-anoxic interface separating the oxic mixolimnion from the anoxic monimolimnion. Samples from 2006, 2008 and 2013–2015 were collected in summer (red crosses), winter (blue circles) and spring (green triangles). Salinity, lake temperature, sunlight and dissolved oxygen measurements based on published data [22, 25]; note that parameters vary with year and season, and data typical for summer are shown. Sunlight, depicted here as Log_e_ of photosynthetically active radiation (μmol m^−2^ s.^−1^), penetrates up to 12 m in summer but only 2 m in winter [22]. **b** Stacked bar chart showing the relative abundance of the *Synechococcus*-like OTU in metagenome data from various lake depths (mixolimnion: surface, oxic 1, oxic 2; oxic-anoxic interface: interface; monimolimnion: anoxic 1, anoxic 2, anoxic 3), seasons (summer, red font: Dec, Jan, Feb; winter, blue font: Jul, Aug; spring, green font: Oct, Nov) and filter fractions (3–20 μm, light green bars; 0.8–3 μm, dark green bars). Data for the 0.1–0.8-μm filter fraction are not shown as the *Synechococcus*-like OTU relative abundances were minimal (≤ 0.3%) [25]. **a** and **b** The images of Vestfold Hills and Ace Lake, and *Synechococcus*-like OTU relative abundances, were modified from [25]. The image of Antarctica was taken from Google Earth (Image Landsat/Copernicus; Image US Geological Survey; US Dept. of State Geographer; Data SIO, NOAA, US Navy, NGA, GEBCO)

In Antarctica, the polar sunlight cycle produces periods of 24-h sunlight in summer and 24-h darkness in winter. Despite the continuous availability of sunlight in summer, metagenomic analyses have determined that the abundance of *Synechococcus* is low or undetectable in the Southern Ocean south of the polar front [19–21]. In contrast, in marine-derived Ace Lake (Vestfold Hills, East Antarctica), a cyanobacterium related to *Synechococcus* (hereafter referred to as *Synechococcus*-like) can bloom to high levels [22–25]. Ace Lake is stratified (meromictic) with a mixed upper oxic zone (mixolimnion) and a stagnant bottom anoxic zone (monimolimnion) that are separated by an oxic-anoxic interface (Fig. 1a) [22, 25–27]. At the oxic-anoxic interface, below the depth at which cyanobacteria bloom, another phototroph, *Candidatus Chlorobium antarcticum* (green sulphur bacteria), thrives, producing considerable biomass (e.g. > 10^8^ cell ml^−1^). The predominance of these two types of phototrophs illustrates the importance that sunlight energy can play in sustaining specific Antarctic, microbially dominated ecosystems [22–25, 28, 29].

As for other *Synechococcus* species, studying the ecophysiology of Ace Lake *Synechococcus*-like species has proved challenging, with cultivation and isolation attempts not achieving axenic cultures; however, a non-axenic culture was obtained and extensively characterised [23]. Subsequent cultivation of this non-axenic culture and DNA sequencing resulted in a genome sequence for *Synechococcus* sp. CS-601 (SynAce01), enabling adaptive traits to be inferred based on comparative genomics [30].

Phylogenetic analyses have placed the Ace Lake cyanobacterium in Marine cluster 5.2 with *Synechococcus* sourced from the water column of neighbouring lakes in the Vestfold Hills: Lake Abraxas and Pendant Lake [23, 30]. *Synechococcus*-like cyanobacterial species have also been reported from other Antarctic aquatic and terrestrial environments, including microbial mats from Highway Lake in the Vestfold Hills, Firelight Lake in the Bølingen Islands [31] and Lake 59b, Lake Reid and Heart Lake in the Larsemann Hills [32, 33]; lakes in northern Victoria Land [34]; the littoral zone of Lundström Lake in the Shackleton Range [35]; lithic substrates from McKelvey Valley, McMurdo Dry Valleys [36]; and weathered granite from Miers Valley, McMurdo Dry Valleys [37].

Expeditions to retrieve samples from Ace Lake for metaproteogenomics first occurred in the austral summer 2006/2007, and subsequently in 2008/2009, with samples covering a complete seasonal cycle obtained in 2013–2015; biomass was collected by sequential size fractionation onto 3.0-, 0.8- and 0.1-μm pore-sized filters, with the metagenome data generated enabling the characterisation of diverse lake microorganisms [24, 25, 28, 29, 38, 39]. The abundance of *Synechococcus*-like operational taxonomic units (OTUs) was assessed according to filter fraction, lake depth and season (Fig. 1b) [24, 25]. *Synechococcus*-like OTUs were present primarily in the 3–20 and 0.8–3 μm filter fractions and were detected at all lake depths sampled; a fermentative capacity was inferred to enable the Ace Lake *Synechococcus*-like species to persist (in low abundance) in the dark, anoxic zone and throughout the water column during periods when sunlight is absent (e.g. winter) [25]. Highest *Synechococcus*-like OTU abundance occurred in the oxic zone in summer (≤ 58% of OTUs in a metagenome), with levels reduced in early winter (≤ 6%) and re-established in late winter (≤ 16%) and spring (≤ 51%); the seasonal abundance dynamics were linked to changes in temperature and available sunlight [23, 25].

To date, genomic variation of Ace Lake *Synechococcus*-like species has not been investigated. However, variation may conceivably occur in response to changes in depth which affects dissolved oxygen (DO) concentration, salinity (increases with depth below the oxic-anoxic interface) and sunlight (decreases with depth and does not penetrate the oxic-anoxic interface) (Fig. 1a), as well as in response to microbial interactions, including with viruses. To assess this, metagenomic reads were mapped to *Synechococcus*-like metagenome-assembled genomes (MAGs) enabling single nucleotide polymorphisms (SNPs), indels (insertion/deletion of multiple bases) and variable coverage regions (VCRs) to be assessed; an approach that has proven successful for studies of other Antarctic species including haloarchaea and *Ca*. Chlorobium antarcticum [29, 40–43]. Here, we interrogated variation of two Ace Lake *Synechococcus*-like MAGs that had different 16S rRNA gene sequences (one each from the oxic zone and anoxic zone) by using Ace Lake metagenomes representing a time and depth series to (i) investigate genomic variation in the *Synechococcus*-like species populations from different seasons (summer vs winter vs spring) and lake depths (oxic vs oxic-anoxic interface vs anoxic) to identify potential phylotypes and ecotypes, (ii) analyse defence genes and potential viral predators of the *Synechococcus*-like species to understand their host-virus interactions and (iii) evaluate the potential factors that might have driven the development of the *Synechococcus*-like species ecotypes.

## Results and discussion

### Overview of Ace Lake metagenomes and *Synechococcus*-like MAGs

A total of 120 time-series Ace Lake metagenomes from summer (Jan, Feb, Dec), winter (Jul, Aug) and spring (Oct, Nov), sampled from seven lake depths (surface, oxic 1, 2 and 3, interface, and anoxic 1, 2 and 3) and 11 time periods (spanning 2006, 2008 and 2013–2015), were used for analyses (Fig. 1a; Additional file 1: Table S1). For fragment recruitment (FR) analysis, 60 metagenomes (302 Gb), in which *Synechococcus*-like OTU relative abundance was ≥ 1% [25], were used to generate 30 merged metagenomes by pooling reads from 3-20 and 0.8–3 μm filter metagenomes that represented specific depths and time periods (Additional file 1: Table S1). For viral analysis, 39,287 Ace Lake viral contigs (724 Mb) from the Antarctic virus catalogue [25, 44] were used to identify potential viruses associated with the *Synechococcus*-like species.

A total of 59 high- or medium-quality MAGs generated from Ace Lake metagenomes (one MAG per metagenome) were analysed (Additional file 2: Dataset S1), of which 25 MAGs were ≥ 99% complete. IMG (Integrated Microbial Genomes) taxonomy classified all the MAGs as SynAce01. Together, the *Synechococcus*-like MAGs consisted of 6681 contigs that encoded 176,198 genes. For FR analysis, two *Synechococcus*-like MAGs were used: one from the Jul 2014, 5 m depth (oxic 1), 3–20 μm filter metagenome (MAG-AL1), and one from the Dec 2014, 14 m depth (anoxic 1), 3–20 μm filter metagenome (MAG-AL2). MAG-AL1 contained 64 contigs, 2929 genes, was 99.7% complete (2,644,322 bp) with 0.09% contamination and was selected for its high bin completeness and lowest bin contamination. MAG-AL2 contained 120 contigs, 2956 genes, was 97% complete (2,654,228 bp) with 0.63% contamination and was selected because it contained a distinct 16S rRNA gene sequence (Additional file 1: Table S2; Additional file 2: Dataset S1).

### *Synechococcus*-like species phylotypes in Ace Lake

**Fig. 2:**
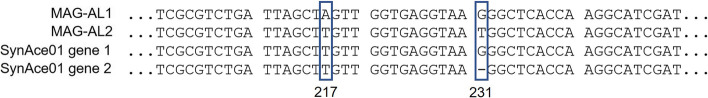
*Synechococcus*-like species 16S rRNA SNP markers. Mismatches at positions 217 and 231 highlighted in the full-length genes from MAG-AL1, MAG-AL2 and the two SynAce01 genes (arbitraily assigned ‘gene 1’ and ‘gene 2’). See Additional file 1: Fig. S1a for the full-length alignment of the four genes

A total of 18 full-length (1489 bp) 16S rRNA genes were identified in *Synechococcus*-like MAGs; they were identical except for the MAG-AL2 gene which distinguished it as a separate phylotype from MAG-AL1 (and all other MAGs) by having two SNPs: 217 A-T and 231 G-T (Fig. 2; Additional file 1: Fig. S1a). By recruiting reads from the merged metagenomes to the two reference MAGs (MAG-AL1 and MAG-AL2), the two SNPs at positions 217 and 231 (i.e. the MAG-AL2 transversions) were determined to be present in all Ace Lake merged metagenomes (Additional file 1: Table S3).

The IMG genome of SynAce01 [30] contains two full-length 16S rRNA genes. MAG-AL1 and MAG-AL2 each contain one full-length 16S rRNA gene, and MAG-AL2 contains an additional incomplete 16S rRNA gene (see supplementary text). The ratio between 16S rRNA SNPs median read depth (from 100% identity FR) and the read depth of its respective MAG was ~ 2 (Additional file 1: Fig. S2), indicating that each MAG contained two very similar 16S rRNA genes, similar to SynAce01. By comparison, the read depth ratio for *Ca*. Chlorobium antarcticum, which contains one 16S rRNA gene [29], was ~ 1 (Additional file 1: Fig. S2).

The two 16S rRNAs from SynAce01 are not identical. Both genes had 217 T (as for MAG-AL2), while ‘gene 1’ had 231 G (as for MAG-AL1) and ‘gene 2’ had a base missing at position 231 (possibly an assembly error) (Fig. 2; Additional file 1: Fig. S1a). The read depths for the two SNP markers for gene 1 were different (Additional file 1: Fig. S3f), and only one read in all the metagenome data matched both SNP markers of gene 1. Due to the difficulties of isolating an axenic and non-clonal strain (see above and Ref. [23]), it is possible that the SynAce01 genome represents two or more closely related *Synechococcus-*like strains. In support of this, FR analyses of the original NCBI SRA SynAce01 reads to SynAce01 16S rRNA genes revealed read sequences with either 217 A plus 231 G (as for MAG-AL1) or 217 T plus 231 T (as for MAG-AL2). For all these reasons, the SynAce01 genome was not used for assessments of population variation (further discussion is provided in supplementary text).

**Fig. 3:**
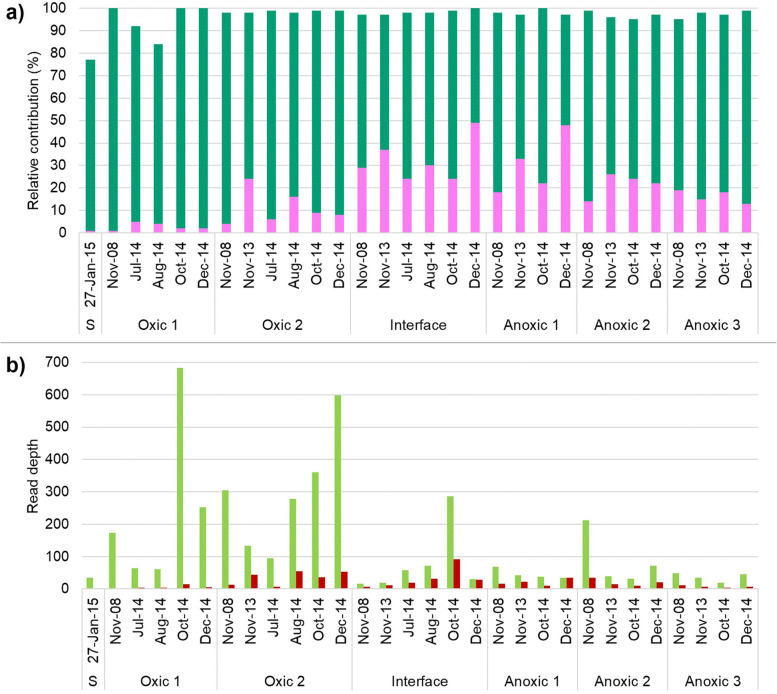
*Synechococcus*-like species phylotype distribution in Ace Lake. **a** Stacked bar chart showing the relative contributions of MAG-AL1 (dark green) and MAG-AL2 (pink) to the overall *Synechococcus*-like species population in each merged metagenome (see Additional file 1: Table S4 for merged metagenome descriptions). MAG contributions were calculated from the frequencies of the specific SNP markers (217 A-T plus 231 G-T). **b** Bar chart showing read depths of MAG-AL1 (light green) and MAG-AL2 (dark red) calculated using their median read depths and percentage contribution. **a** and **b** Merged metagenomes (*x*-axis) include data from metagenomes in which *Synechococcus*-like OTU abundance was ≥ 1% and is first arranged by depth and then time period. Depths: oxic depths, S (surface), oxic 1 and oxic 2; oxic-anoxic interface, interface; anoxic depths, anoxic 1, anoxic 2 and anoxic 3

The SNP markers (217 A-T plus 231 G-T) were used to evaluate the contributions of the MAG-AL1 and MAG-AL2 phylotypes to the total *Synechococcus*-like species population (Fig. 3a; Additional file 1: Table S4). The relative contribution of the two phylotypes varied with depth and season. In all metagenomes, the MAG-AL1 phylotype contributed the most to the *Synechococcus*-like species population, with highest representation in the oxic zone. The MAG-AL2 phylotype had highest representation (almost 50% of the total *Synechococcus*-like species population) in the oxic-anoxic interface or anoxic 1 depth (Fig. 3a).

The relative contributions of MAG-AL1 and MAG-AL2 were used to calculate their abundances in Ace Lake highlighting their distribution throughout the water column (Fig. 3b). The highest abundance of MAG-AL2 in each time period occurred at different depths, with it being prevalent at the oxic-anoxic interface and surrounding depths. This suggested that MAG-AL2 signatures from the anoxic depths might not be from dead cells.

In addition to the two SNPs that define MAG-AL1 and MAG-AL2, seven additional SNPs were identified in three oxic zone metagenomes (Additional file 1: Table S3). These seven SNPs might be indicative of other *Synechococcus*-like species phylotypes (distinct from MAG-AL1 and MAG-AL2) in the oxic zone, particularly in surface waters, where SNP frequencies were higher (21–25%) than in oxic 1 (16–19%) and oxic 2 (3–5%) (Additional file 1: Table S3).

### Phylogeny and global representation of Ace Lake *Synechococcus*-like species

In addition to the high identity between 16S rRNA genes from MAG-AL1 and MAG-AL2 (99.9% identity), the average nucleotide identity (ANI: 99.6% over 92% alignment fraction), average amino acid identity (AAI: 99.3% over 90% alignment fraction) and digital DNA-DNA hybridisation (dDDH: 97%) were high (Additional file 1: Figs. S1 and S4), indicating that these two phylotypes belonged to the same species and subspecies.

**Fig. 4:**
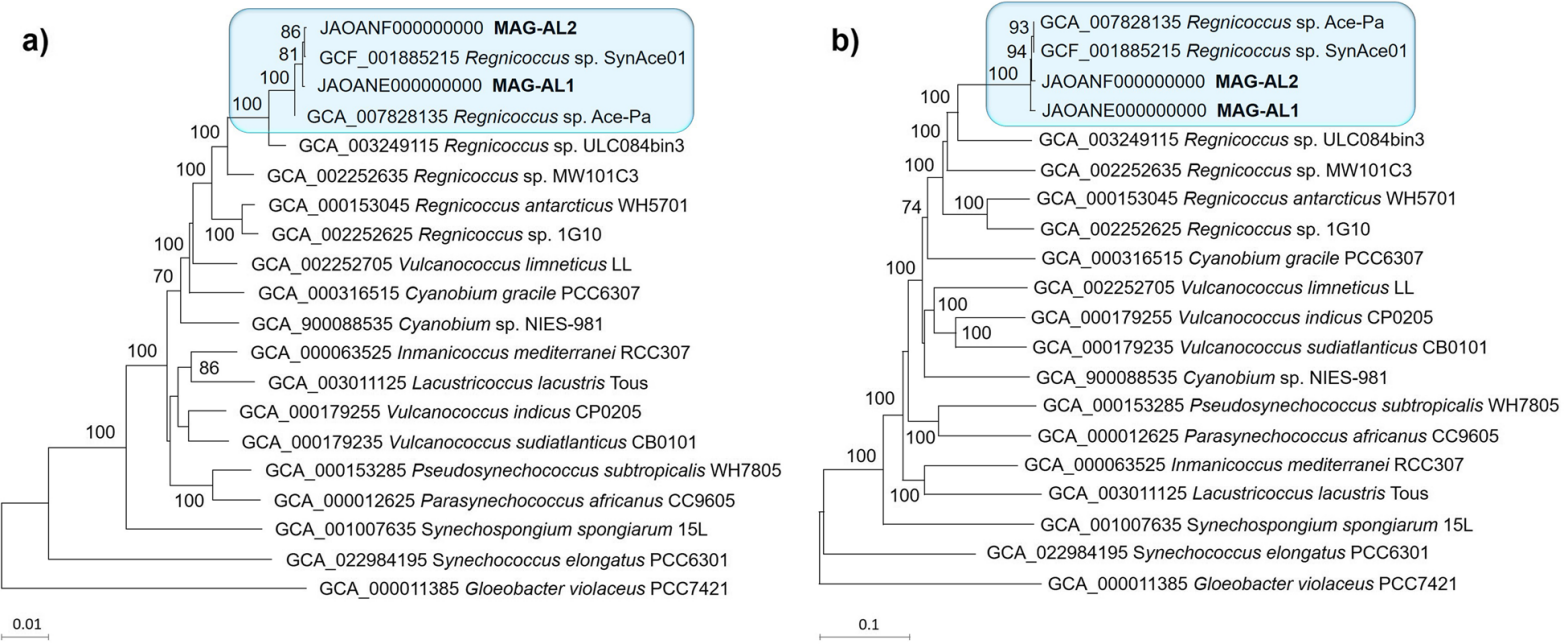
Ace Lake cyanobacterium phylogeny. Minimum evolution trees based on **a** 16S rRNA genes and **b** whole proteomes of MAG-AL1, MAG-AL2 and SynAce01, along with reference genomes and MAGs obtained from GTDB. The 16S rRNA gene sequences of MAG-AL1 and MAG-AL2 were taken from their MAG bins in IMG (MAG-AL1, 3300023237_10; MAG-AL2, 3300023253_6). Trees are drawn to scale, with scale bars indicating branch lengths. The numbers at nodes are Genome BLAST Distance Phylogeny pseudo-bootstrap support values > 70% from 100 replications, with an average branch support of 52.9% (**a**) or 93.6% (**b**). Ace Lake cyanobacteria are highlighted. *Regnicoccus* sp. Ace-Pa (or *Synechococcus* sp. Ace-Pa) is an unpublished, draft genome assembled from the same Ace Lake cyanobacterial isolate as SynAce01 (here *Regnicoccus* sp. SynAce01) and was not used for analyses

Phylogenetic tree construction based on 16S rRNA genes and whole proteome content (Fig. 4), and ANI and AAI analyses of closely related MAGs and reference genomes obtained from GTDB (Additional file 3: Dataset S2) demonstrated that Ace Lake cyanobacterial genomes (SynAce01, MAG-AL1, MAG-AL2) formed a tight clade with ≤ 82% ANI and ≤ 79% AAI to other taxa. The MAG-AL1 and MAG-AL2 16S rRNA genes had ≤ 98% identity to 16S rRNA genes available in databases from IMG publicly assembled metagenomes and public isolates (Additional file 3: Dataset S2). The closest non-Antarctic species (≤ 98% identity) included *Synechococcus* sp. 1G10 (Nahuel Huapia Lake, Argentina), *Synechococcus* sp. MW101C3 (Lake Mondsee, Austria) and *Synechococcus* sp. WH5701 (Long Island Sound, New York; Ref. [45]). Each of these species, along with SynAce01 from Ace Lake, has recently been placed in the novel genus *Regnicoccus* [5]. The 16S rRNA genes of *Synechococcus* from Lake Abraxas and Pendant Lake (Vestfold Hills; Ref. [30]) were ≤ 98% identical to the MAG-AL1 and MAG-AL2 sequences. Based upon these data, the Ace Lake cyanobacterium appears to represent a distinct species that is possibly confined to this water body. This contrasts with the green sulphur bacterium, *Ca.* Chlorobium antarcticum, which has an identical 16S rRNA gene sequence from Ace Lake, Taynaya Bay and Ellis Fjord [29]. In view of the phylogenetic characteristics of the Ace Lake cyanobacterium, we name a new Ace Lake species: *Candidatus* Regnicoccus frigidus sp. nov. (from fri’gi.dus. L. masc. adj. frigidum cold, referring to the cold environment) (type MAG MAG-AL1: GenBank accession ID = JAOANE000000000; IMG bin ID = 3300023237_10; 99.7% complete; 0.09% contamination) (Additional file 2: Dataset S1).

### *Ca.* Regnicoccus frigidus population variation

**Fig. 5:**
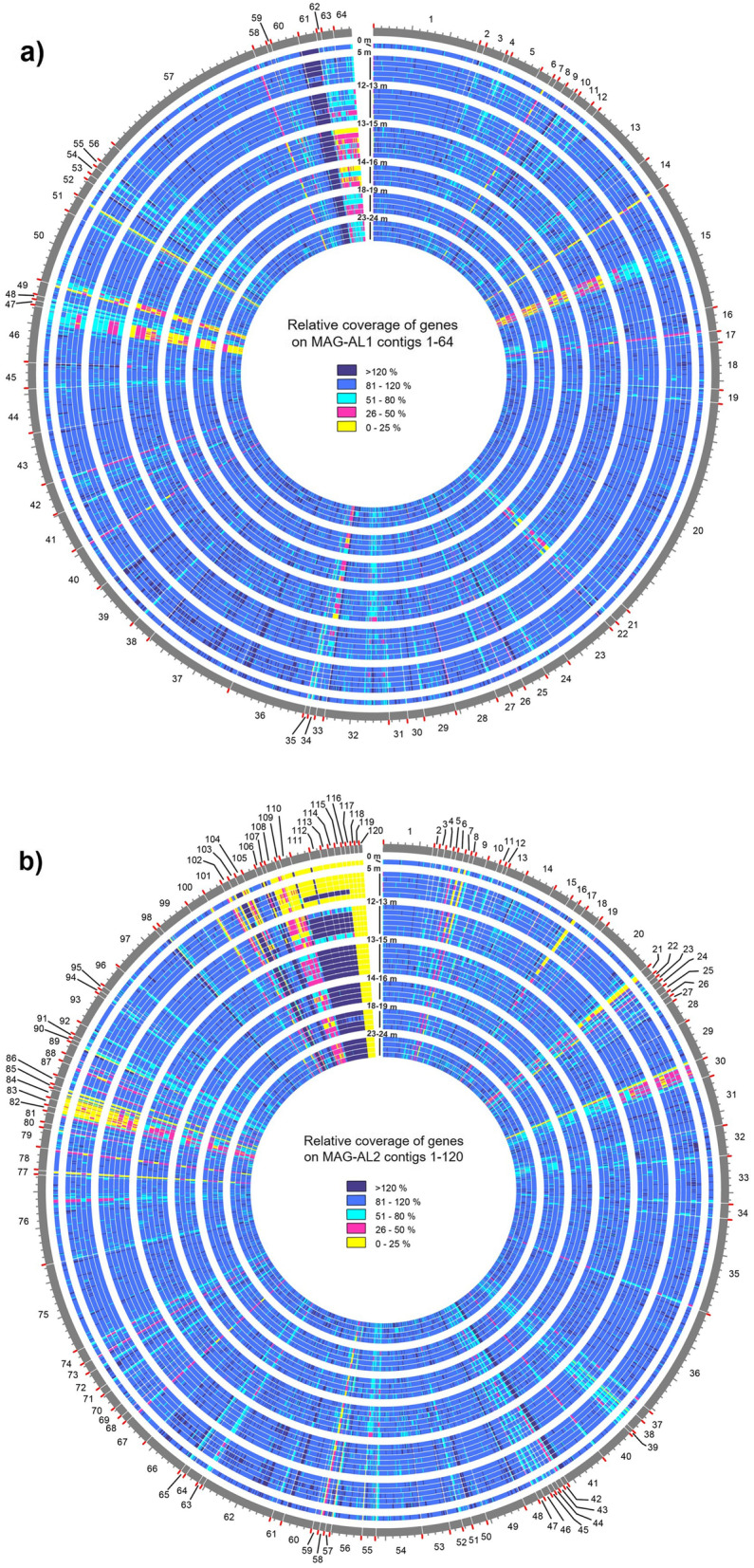
Genomic variation in *Ca.* Regnicoccus frigidus population. Circos plots depict the distribution of relative coverages of genes in **a** MAG-AL1 and **b** MAG-AL2, in merged metagenomes from various Ace Lake depths and time periods (see Additional file 1: Table S4 for merged metagenome descriptions). The *x*-axes are shown on the outermost annuli: beginning of every contig, red tick; 10 kb length, grey tick. The annuli are first segregated by lake depth (surface, 0 m; oxic 1, 5 m; oxic 2, 12–13 m; interface: 13–15 m; anoxic 1, 14–16 m; anoxic 2, 18–19 m; anoxic 3, 23–24 m) and then arranged by season (summer: Jan, Dec; winter: Jul, Aug; spring: Oct, Nov). Metagenome reads with ambiguous mapping were allowed to map to the first best site, resulting in coverages of genes with multiple copies exceeding 100%; for display purposes, coverage was capped at 120%. Outer to inner annuli: MAG-AL1 contigs 1–64 or MAG-AL2 contigs 1–120; 0 m: 27 Jan 2015; 5 m: Dec 2014, Jul 2014, Aug 2014, Nov 2008 and Oct 2014; 12–13 m: Dec 2014, Jul 2014, Aug 2014, Nov 2008, Nov 2013 and Oct 2014; 13–15 m: Dec 2014, Jul 2014, Aug 2014, Nov 2008, Nov 2013 and Oct 2014; 14–16 m: Dec 2014, Nov 2008, Nov 2013 and Oct 2014; 18–19 m: Dec 2014, Nov 2008, Nov 2013 and Oct 2014; 23–24 m: Dec 2014, Nov 2008, Nov 2013 and Oct 2014

SNPs (variant frequency ≥ 0.9), indels (read depth ≥ 20) and VCRs (significance of gene coverage variation assessed using DESeq2) were identified from FR of reads that represented different lake depths and time periods to MAG-AL1 and MAG-AL2 (Fig. 5; Additional file 4: Dataset S3). Variation was lower in MAG-AL1 (75 SNPs and 17 indels from 45 genes) than MAG-AL2 (572 SNPs and 27 indels from 157 genes) (Additional file 4: Dataset S3).

SNPs and indels were identified in genes involved in cell wall or membrane biosynthesis and modification, transport, translation, carbohydrate metabolism, amino acid biosynthesis and other metabolic processes, as well as some hypothetical genes (Additional file 4: Dataset S3). Only a few SNPs and indels from each MAG were consistently represented across metagenomes from different time periods of the same depth (Additional file 4: Dataset S3; further discussed in supplementary text). This indicated that most *Ca.* Regnicoccus frigidus mutations were not stable, with the temporal variation observed being indicative of a relatively dynamic population. The mutations in MAG-AL1 and MAG-AL2 genes were observed mainly in the anoxic and oxic depth metagenomes, respectively (Additional file 4: Dataset S3). MAG-AL1 genes containing stable mutations included the following: a glycosyltransferase (cell wall modification) and 2-oxoisovalerate dehydrogenase (branched-chain amino acids degradation) (Additional file 4: Dataset S3). MAG-AL2 genes with stable mutations encoded the following: carboxysome shell carbonic anhydrase (carbon dioxide fixation); a vitamin K epoxide reductase family protein (post-translational modification); glycerol-3-phosphate acyltransferase (glycerolipid synthesis); N-acetylglucosamine-6-phosphate deacetylase (cell wall synthesis and glycolysis); a glycosyltransferase (cell wall modification); UDP-glucuronate decarboxylase (cell wall modification); and four hypothetical proteins (Additional file 4: Dataset S3).

**Fig. 6:**
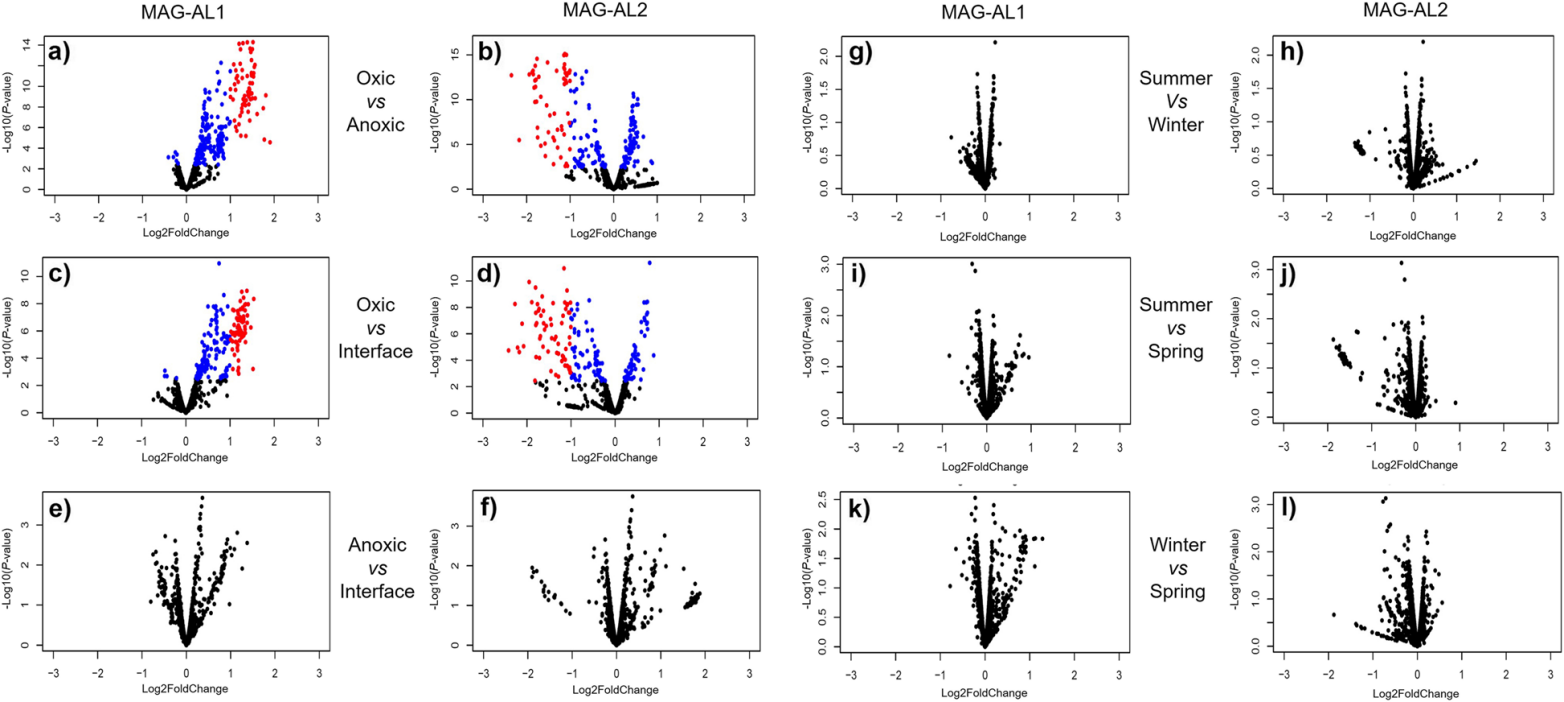
Depth-dependent and seasonal variations in gene coverages of *Ca.* Regnicoccus frigidus MAGs. Volcano plots showing variations in MAG-AL1 (**a**, **c**, **e**, **g**, **i**, **k**) and MAG-AL2 (**b**, **d**, **f**, **h**, **j**, **l**) gene coverages in samples from different lake depths (oxic, interface, anoxic) or seasons (summer, winter, spring). Each dot in a plot represents a MAG gene. The *y*-axes indicate the statistical significance (− log10 of *P*-value) of the change in gene coverage, with significance increasing from bottom to top: red dots, genes with fold change > 1 or <  − 1 and *P* ≤ 0.05; blue dots, genes with fold change between − 1 and 1 and *P* ≤ 0.05; black dots, genes with *P* > 0.05. The *y*-axis scale varies for each plot to enhance data visibility. See Additional file 4: Dataset S3 for a list of genes with significant fold changes. The *x*-axes indicate the magnitude of change (log2 of fold change) in gene coverage: positive fold change, gene coverage higher in samples taken as numerator; negative fold change, gene coverage higher in samples taken as denominator. For example, in the oxic vs anoxic plots (**a**) and (**b**), the oxic zone samples were considered as numerator, while the anoxic zone samples were taken as denominator. Therefore, the MAG-AL1 genes represented by red dots in **a** have significantly higher relative coverage in oxic zone samples, whereas the MAG-AL2 genes depicted by red dots in **b** have significantly higher relative coverage in anoxic zone samples. **g**–**l** No significant gene coverage variations were observed during comparison of seasonal samples (see further description in supplementary text)

Most variable coverage genes (VCGs) with significant coverage variation were of unknown function, i.e. hypothetical or uncharacterized proteins, poorly characterized or coded for mobile elements (Additional file 4: Dataset S3). The remainder were genes involved in the following: cell wall or membrane biosynthesis and modification, transport, stress response, cell defence, cyanide assimilation and other metabolic functions (Additional file 4: Dataset S3). Significant gene variations were only identified for comparisons by depth, specifically oxic vs anoxic and oxic vs oxic-anoxic interface (Fig. 6; Additional file 4: Dataset S3). Most VCGs with similar function had distinct sequences in MAG-AL1 and MAG-AL2, with the depth-dependent variation specific to each MAG: the VCGs had higher coverage in the oxic zone for MAG-AL1 and higher coverage in the oxic-anoxic interface and anoxic zone for MAG-AL2 (Figs. 5 and 6; Additional file 4: Dataset S3). This pattern of coverage matched phylotype abundance, with MAG-AL1 more prevalent in the oxic zone and MAG-AL2 more so in the oxic-anoxic interface and surrounding depths (Fig. 3b; Additional file 1: Table S4). These data would be consistent with niche adaptation, with MAG-AL1 and MAG-AL2 possessing genetic capacities tailored to growth and survival in the oxic and anoxic zones, respectively (also see below in ‘Niche adaptation in Ace Lake’).

Alignments of *Ca.* Regnicoccus frigidus MAGs revealed that contigs that did not align or had poor alignment tended to contain VCGs or putative viral genes (Additional file 5: Dataset S4). However, MAG-AL2 contigs 118–120 did not match any other MAGs. These contigs had low read depth in all metagenomes, and their gene relative coverages showed depth-dependent variation: oxic-anoxic interface and anoxic zone, < 24%, and oxic zone, ≤ 0.2% (Additional file 4: Dataset S3). Some of the genes on these contigs (e.g. glycine hydroxymethyltransferase, ATP adenylyltransferase, bifunctional demethylmenaquinone methyltransferase/2-methoxy-6-polyprenyl-1,4-benzoquinol methylase UbiE, murein DD-endopeptidase MepM/murein hydrolase activator NlpD, MFS family permeases, selenophosphate synthetase) were present with normal coverage elsewhere in MAG-AL2, suggesting that all *Ca.* Regnicoccus frigidus populations studied possessed these functional traits.

The remaining low coverage genes represented *Ca.* Regnicoccus frigidus populations at the oxic-anoxic interface and in the anoxic zone that possessed a genetic capacity not present in the *Ca.* Regnicoccus frigidus populations in the oxic zone. Two genes annotated as a carbon monoxide dehydrogenase (CODH) maturation factor and a predicted RNA-binding protein contained CooC and CooT domains, respectively; these are domains found in accessory proteins involved in the maturation of anaerobic CODH that occurs by the insertion of nickel into the active site [46, 47]. No CODH genes were identified in *Ca.* Regnicoccus frigidus MAGs, suggesting that the CooC and CooT domain-containing enzymes may function in anaerobic process(es) involving nickel-dependent pathways.

GC content of contigs was plotted against read depth to assess the presence of contig clusters representative of divergent (< 95% sequence similarity) *Ca.* Regnicoccus frigidus phylotypes (Additional file 1: Fig. S5). Taxonomic analysis of 51,971 metagenome contigs adjacent to or overlapping the *Ca.* Regnicoccus frigidus MAG contigs (i.e. metagenome contigs with 45–80% GC content) revealed that only 297 metagenome contigs were classified as Cyanobacteria, and many of these had ≥ 99% identity matches to assembled *Ca.* Regnicoccus frigidus MAGs (Additional file 1: Table S5; also see supplementary text). The analyses indicate that *Ca.* Regnicoccus frigidus phylotypes with a high level of divergence (< 95% sequence similarity) were not abundant and, given the large size of the Ace Lake dataset, are not typical of the lake ecosystem.

### *Ca.* Regnicoccus frigidus viruses

*Ca.* Regnicoccus frigidus viral contigs were identified in several ways (Additional file 6: Dataset S5): A) 31 in IMG/VR v3 from Ace Lake metagenomes: vOTU_081954 (22), vOTU_248451 (7), Sg_292136 (1) and Sg_613705 (1). B) 22 viral contigs aligned to the 59 *Ca.* Regnicoccus frigidus MAGs: vOTU_022592 (16) and Sg_256402 (1) from IMG/VR v3 and cl_2442 (2), cl_463 (1), sg_14817 (1) and sg_14822 (1) from the Antarctic virus catalogue. C) 11 previously identified viral contigs based on matches to a cyanophage assembled from Ace Lake metagenome data: cl_6580 (2), cl_6727 (2), cl_9495 (1), cl_9892 (1), sg_14929 (1), sg_14949 (1), sg_14969 (1), sg_14971 (1) and sg_15003 (1) from the Antarctic virus catalogue [25]

The set of 22 and the set of 11 viral contigs contained genes that were taxonomically classified to a variety of microorganisms, indicating the viral contigs might represent generalist viruses that prey on multiple hosts. Five of the 22 viral contigs were present in two *Ca.* Regnicoccus frigidus MAGs, two verrucomicrobial MAGs and one actinobacterial MAG (Additional file 6: Dataset S5), indicating that these viral contigs likely represented prophages in the respective MAGs. The set of 31 viral contigs included three predicted prophages in IMG/VR v3 (Additional file 6: Dataset S5). Two of these prophages plus 10 other viral contigs aligned to some *Ca.* Regnicoccus frigidus MAGs (Additional file 6: Dataset S5). The prediction of prophages is consistent with two prophage regions (phiSynAce1 and phiSynAce2) reported for the SynAce01 genome [30].

Overall, the data for these three sets of viral contigs suggests that (i) vOTU_081954, vOTU_248451, Sg_292136 and Sg_613705 represent *Ca*. Regnicoccus frigidus specialist viruses, some of which are prophages; (ii) Sg_717548 and Sg_723842 (and nine viral contigs from the Antarctic virus catalogue) represent generalist viruses that prey on cyanobacteria; and (iii) Sg_256402 and vOTU_022592 (and five viral contigs from the Antarctic virus catalogue) likely represent generalist viruses that prey on an even broader range of hosts (Additional file 6: Dataset S5).

MAG-AL2 contained more predicted prophages than MAG-AL1, although the total viral gene composition for each MAG was similar (Additional file 6: Dataset S5). Gene coverage of the predicted MAG prophages was high (MAG-AL1 ≤ 7000 read depth; MAG-AL2 ≤ 8000) compared to MAG read depths (both MAGs < 700) (Additional file 6: Dataset S5). The set of 31 *Ca.* Regnicoccus frigidus viral contigs had high coverage (< 6500), some of which belonged to *Ca.* Regnicoccus frigidus MAGs. The high coverage contigs are likely to represent viral progeny of integrated (i.e. MAG prophage) or nonintegrated viruses associated with cells.

Searches for additional prophages in MAG-AL1 and MAG-AL2 were performed based on read coverage (i.e. high), gene composition and/or proximity to already predicted prophages. All prophages identified by this process were ≤ 19 kb in length, which is short by comparison to known cyanophages and therefore likely represent remnants of previous prophages (Additional file 6: Dataset S5). The MAG-AL2 prophage genes on contigs 17, 106 and 111–116 had very low relative coverages in surface and oxic 1 (except Nov 2008) metagenomes compared to metagenomes from deeper depths (Additional file 6: Dataset S5), possibly reflecting a greater loss of these prophage genes from the *Ca.* Regnicoccus frigidus population in the upper waters of the lake.

### Host defence against viruses

Restriction-modification (RM) systems can be encoded by hosts and/or viruses and can impact host-virus interactions in a variety of ways [41, 48–50]. The prophages within MAG-AL1 and MAG-AL2 contained a type 2 RM DNA methylase, with MAG-AL2 prophages also containing two type 1 RM DNA methylases (Additional file 6: Dataset S5). Type 2 RM methyltransferases have been associated with lysogenic lifestyles [49], which would be consistent with the prophage remnants arising from an integrated temperate virus. All subunit genes of a type 1 RM system, and additional genes associated with RM systems (e.g. type 3 Res subunit domain), were also present outside of the prophages in MAG-AL1 and MAG-AL2 and were therefore host-specific RM genes (Additional file 7: Dataset S6).

CRISPR-Cas system genes were not identified in *Ca.* Regnicoccus frigidus MAGs, consistent with previous findings for Ace Lake *Synechococcus*-like OTUs [25] and other marine cyanobacteria [51]. However, systems potentially involved in host-virus interactions included the DISARM (defence island system associated with restriction-modification) and retron systems. DISARM genes identified in *Ca.* Regnicoccus frigidus MAGs were *drmMII* (DNA [cytosine-5]-methyltransferase) and *drmD* (SNF2 family DNA/RNA helicase), although genes constituting a complete system were not identified (Additional file 7: Dataset S6).

Retrons are often found near defence systems such as RM genes and afford viral defence via an ABI (abortive infection) mechanism and have previously been identified in cyanobacteria [52]. Bacterial retrons consist of a reverse transcriptase gene, a noncoding RNA and an effector gene which encodes a DNA-binding, HNH endonuclease, ribosyltransferase or two transmembrane (2TM) domains [52]. A reverse transcriptase gene containing a bacterial retron domain was identified close to a type 1 RM system in MAG-AL1 (Additional file 1: Fig. S6; Additional file 7: Dataset S6). Most genes near the retron homologue were hypothetical genes and did not match known retron effector domains (Additional file 1: Fig. S6), although exostosin family protein domain (TM domain) and HicB antitoxin (which contains HTH domain; Refs. [53, 54]) genes were identified adjacent to retron homologs in *Ca.* Regnicoccus frigidus MAGs and could possibly function as effector genes to constitute a functional retron anti-phage system.

**Fig. 7:**
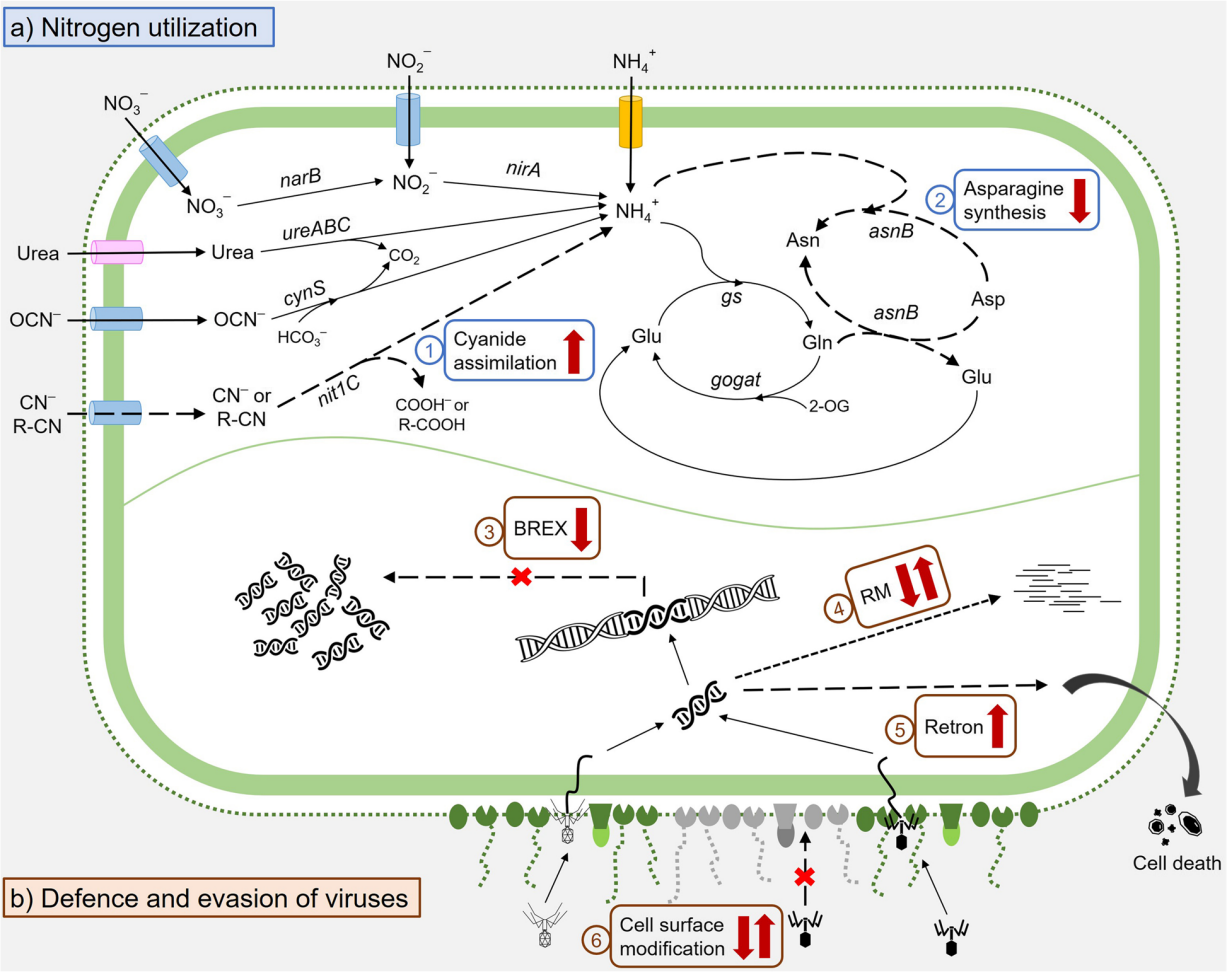
*Ca.* Regnicoccus frigidus niche adaptation in Ace Lake. **a** *Ca.* Regnicoccus frigidus ecotypes with the capacity for (1) cyanide assimilation and/or (2) asparagine biosynthesis: yellow cylinder, ammonium transporter; blue cylinders, nitrate/nitrite transporters; pink cylinder, urea transporter. **b** Variability in the capacity of *Ca.* Regnicoccus frigidus populations to defend against or evade viruses: (3) BREX type 1 system, (4) type 1 RM system, (5) retron and (6) cell surface modification: green shapes, cell surface structures; grey shapes, modified cell surface structures; black virions, host-specific (specialist) viruses; light grey virions, generalist viruses. Genes found in all *Ca.* Regnicoccus frigidus (solid line arrows), *Ca.* Regnicoccus frigidus subpopulations (dashed line arrows) or all *Ca.* Regnicoccus frigidus plus a subpopulation containing additional genes (dotted line arrows). Genes identified in *Ca.* Regnicoccus frigidus subpopulations with depth-dependent coverage variation (dark red arrows): up, higher coverage in oxic zone; down, higher coverage in oxic-anoxic interface and anoxic zone; both up and down, coverage high in either zone due to distinct gene sequences in MAG-AL1 and MAG-AL2. Genes and substrates: Asn, asparagine; *asnB*, glutamine-hydrolysing asparagine synthetase; Asp, aspartate; BREX, bacteriophage exclusion; CN^−^, free cyanide; CO_2_, carbon dioxide; COOH^−^ or R-COOH, carboxylic acid compounds; *cynS*, cyanate lyase; Gln, glutamine; Glu, glutamate; *gogat*, ferredoxin-glutamine oxoglutarate aminotransferase; *gs*, glutamine synthetase; HCO_3_^−^, bicarbonate; *narB*, ferredoxin-nitrate reductase; NH_4_^+^, ammonium; NO_2_^−^, nitrite; NO_3_^−^, nitrate; *nirA*, ferredoxin-nitrite reductase; *nit1C*, nitrilase from Nit1C gene cluster; OCN.^−^, cyanate; 2-OG, 2-oxoglutarate; R-CN, nitriles; *ureABC*, urease (alpha, beta, gamma subunits). Virions, viral and host DNA and degraded cell icons were taken from The Noun Project website [55]

Depth-dependent variation was observed for some of the above *Ca.* Regnicoccus frigidus defence genes (Additional file 7: Dataset S6). The MAG-AL1 retron homolog, *drmMII*, type 1 RM system (two S, one R and one M subunit) and three putative RM (two Uma2 family endonucleases and one HNH family endonuclease) genes had higher coverage in the oxic zone than in the oxic-anoxic interface or anoxic zone (Fig. 7; Additional file 4: Dataset S3; Additional file 7: Dataset S6). A similar pattern of variation occurred for MAG-AL2 *drmMII*, type 1 RM S subunit and putative RM (HNH family endonuclease) genes. Viruses are prevalent throughout Ace Lake, but abundance is highest in the oxic zone [25]. These aforementioned systems that are overrepresented in the oxic zone may reflect a functionality particularly suited to responding to the specific viral population. In contrast, two type 1 RM R subunit genes and a putative RM gene (Uma2 family endonuclease) that were specific to MAG-AL2 had 2 to 3 times higher coverage in the oxic-anoxic interface and anoxic zone than the oxic zone (Additional file 4: Dataset S3; Additional file 7: Dataset S6). As MAG-AL2 is prevalent in the anoxic zone, the higher coverage for these defence systems suggests they are more specific to viruses enriched in the anoxic zone (Fig. 3; Additional file 7: Dataset S6).

Bacteriophage exclusion (BREX) type 1 system genes (*brxC*, *brxB*, *brxA* and truncated *pglX* and *brxL*) and additional BREX genes (*brxHI*, *brxHII*, *pglW*) were identified in both MAG-AL1 and MAG-AL2, and *pglZ* and complete *brxL* (often together) were present in some other *Ca.* Regnicoccus frigidus MAGs (Additional file 1: Fig. S6; Additional file 7: Dataset S6). The *pglX* gene was truncated in MAG-AL1 and MAG-AL2, and some *Ca.* Regnicoccus frigidus MAGs contained two truncated *pglX* genes that together constituted the full-length gene (Additional file 1: Fig. S6; Additional file 7: Dataset S6; further discussed in supplementary text). A complete *pglX* cyanobacterial gene was also identified in Ace Lake contigs. Similar observations were made for Antarctic haloarchaea resident in Deep Lake (Vestfold Hills) [41]. Interruption of the *pglX* gene occurs in a diverse range of microorganisms with acquisition of the gene-by-gene transfer enabling the BREX system to be functional [56]. In addition to the variation in the integrity of the *pglX* gene, only a subset of *Ca.* Regnicoccus frigidus MAGs contained complete sequences of *brxL* and *pglZ* (core BREX gene) (Additional file 7: Dataset S6). Using a MAG that contained *brxL* and *pglZ* (99.7% bin completeness, 3.6% bin contamination, Dec 2014, 12 m depth, 0.8-μm filter metagenome), both genes were found to have low relative coverages (≤ 25%) in all metagenomes, with coverage significantly higher in the oxic-anoxic interface and anoxic zones than in the oxic zone (Additional file 4: Dataset S3). These data show depth-dependent variation for BREX genes, with less than a quarter of the *Ca.* Regnicoccus frigidus population possessing *brxL* and *pglZ*; that subpopulation would also need to possess (vertical inheritance) or acquire (gene transfer) a functional *pglX* gene in order to perform BREX-mediated viral resistance (Fig. 7). It therefore appears that the *Ca.* Regnicoccus frigidus population is limited in its ability to mount, at best, a transient BREX response.

### Host evasion of viruses

Variation (VCRs, SNPs, indels) was a feature of a variety of genes encoding cell surface proteins (e.g. TolC and porins) or genes involved in cell wall biosynthesis (e.g. lipopolysaccharides) and modification (e.g. glycosyltransferases) (Additional file 4: Dataset S3). Viruses attach to cell surface components, including lipopolysaccharides, TolC and porins [57]. The coverages of these VCGs involved in cell surface structures in MAG-AL1 were significantly higher in the oxic zone than in the oxic-anoxic interface or anoxic zone, while the opposite trend occurred for MAG-AL2 (with the exception of a few glycosyltransferases). Moreover, the specific VCGs in MAG-AL1 differed to those in MAG-AL2, suggesting that the cell wall composition of the *Ca.* Regnicoccus frigidus represented by the two MAGs was likely to differ (Additional file 4: Dataset S3).

SNPs and indels identified in some of the *Ca.* Regnicoccus frigidus glycosyltransferases (Additional file 4: Dataset S3) may impact cell wall composition by influencing substrate specificity of the enzymes and the type of sugar they incorporate during glycosylation [58, 59]. Variation in glycosyltransferases and other cell surface proteins has been speculated to mediate viral evasion in Antarctic haloarchaea [41, 43] and marine *Prochlorococcus* [60], and liposaccharide modification has been shown to perturb viral infection of *Anabaena* sp. PCC7120 [61]. The types of genetic variation observed for *Ca.* Regnicoccus frigidus is therefore likely a response to interactions with viruses, particularly as a mechanism of evasion of specialist viruses that target specific epitopes during viral attachment (Fig. 7).

### Niche adaptation in Ace Lake

Specific relationships were evident between *Ca.* Regnicoccus frigidus phylotype abundances and physicochemical data (Fig. 3; Additional file 1: Table S4). Significant correlations occurred between changes in MAG-AL1 abundance and depth (Spearman’s rank correlation coefficient *ρ* =  − 0.6, *P* = 0.003), DO (*ρ* = 0.5, *P* = 0.008) and salinity (*ρ* =  − 0.6, *P* = 0.003), but not lake temperature (*ρ* =  − 0.4, *P* = 0.1). In contrast, no significant correlations occurred with MAG-AL2 abundance. Of these lake factors, salinity has previously been associated with the evolution of *Synechococcus* and *Prochlorococcus* ecotypes in the South China Sea [17].

Significant depth-dependent variation in MAG-AL1 and MAG-AL2 gene coverages was observed for oxic vs anoxic and oxic vs oxic-anoxic interface metagenomes (Fig. 6; Additional file 4: Dataset S3). The functional properties of the VCGs encoding metabolic functions were examined to assess what ecophysiological impact they may confer.

#### Cyanide assimilation

**Fig. 8:**
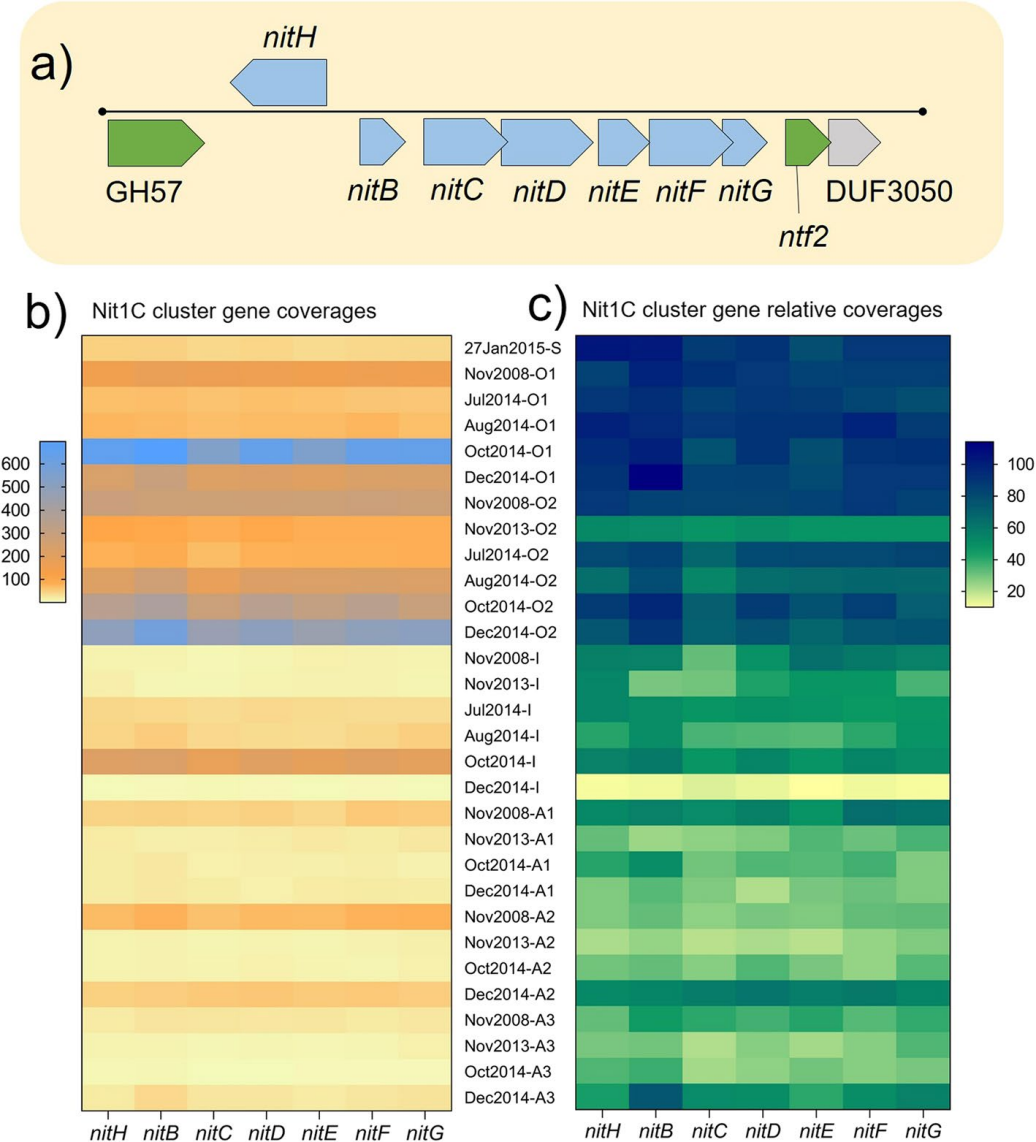
Depth profile of *Ca.* Regnicoccus frigidus Nit1C gene cluster. **a** Schematic of Nit1C gene cluster on contig 33 (8931 bp length) of MAG-AL1: blue, Nit1C cluster genes; green, other metabolic genes; grey, uncharacterised gene; black line, contig backbone with black dots representing contig ends. **b** and **c** Heat maps showing coverages (**b**) and relative coverages (**c**) of Nit1C cluster genes in Ace Lake merged metagenomes. Gene relative coverages (%) indicate the proportion of *Ca.* Regnicoccus frigidus population in each merged metagenome that contained the gene. Data arranged from top to bottom by depth followed by time period of sample collection. Depths: S, surface; O1, oxic 1; O2, oxic 2; I, interface; A1, anoxic 1; A2, anoxic 2; A3, anoxic 3. Genes: DUF3050, DUF3050 domain-containing protein; GH57, glycosyl hydrolase family 57 protein; *nitB*, nitrogen starvation response protein; *nitC*, nitrilase; *nitD*, radical SAM superfamily protein; *nitE*, GNAT family N-acetyltransferase; *nitF*, AIR synthase-like protein; *nitG*, nitrogen starvation response protein; *nitH*, flavin-dependent oxidoreductase; *ntf2*, nuclear transport factor 2 family protein

A Nit1C gene cluster (*nitHBCDEFG*; contig 33) was identified as a VCR in MAG-AL1, but not in MAG-AL2 (Fig. 8a; Additional file 4: Dataset S3). This locus, which has previously been reported in cyanobacteria, belongs to branch 1 nitrilases that can function during nitrogen starvation to assimilate nitriles by hydrolysing them to ammonia (plus a carboxylic acid) [62–67]. Nit1C gene expression can be highly induced by cyanide and repressed by ammonium and is essential for growth when cyanide is the sole source of nitrogen [64, 65, 67].

The Nit1C cluster had significantly higher coverage (*P* ≤ 0.0002) in the oxic zone (81%), compared to the oxic-anoxic interface (41%) or anoxic zone (36%) (Fig. 8b and c; Additional file 4: Dataset S3). The littoral mats in Ace Lake contain diverse cyanobacteria as well as predatory ciliates and rotifers [22, 26], and the ability to produce cyanide is widespread among phylogenetically diverse cyanobacteria, possibly as a defence mechanism against grazers (e.g. ciliates, rotifers) [68]. It is therefore possible that the Ace Lake cyanobacteria in the littoral mats generate relatively high levels of free cyanide (HCN and CN^−^) in the oxic zone, with cyanate generated by abiotic cyanide oxidation. The lower Nit1C cluster coverage in the anoxic zone is consistent with this zone having relatively high levels of ammonium (which represses gene expression) [22, 64, 69, 70]. Cyanate transporter genes were not identified in MAG-AL1, but nitrate and nitrite transporters which were encoded could possibly function in the uptake of cyanate and cyanide [71, 72]. These data would be consistent with MAG-AL1 Nit1C genes being induced during nitrogen starvation and/or in the presence of cyanide, allowing *Ca.* Regnicoccus frigidus to assimilate free cyanide and nitriles as nitrogen sources. As bioavailable nitrogen is limiting in the oxic zone [22, 70], the Nit1C gene cluster would be expected to enhance the competitiveness of the *Ca.* Regnicoccus frigidus population that possess it.

#### Asparagine synthesis

The conversion of aspartate to asparagine can be catalysed by AsnB (glutamine-hydrolysing asparagine synthetase) using glutamine as the preferred substrate or ammonium [73]. An *asnB* gene (IMG gene ID: Ga0222690_10005105) was identified in 18 *Ca.* Regnicoccus frigidus MAGs (but not in MAG-AL1 or MAG-AL2). While the ammonium-dependent asparagine synthetase gene (*asnA*) was not identified in *Ca.* Regnicoccus frigidus MAGs, the capacity to use nitrate, nitrite and ammonia for glutamine production via the GS-GOGAT (glutamine synthetase-glutamate synthase) cycle was evident in *Ca.* Regnicoccus frigidus (Fig. 7).

**Fig. 9:**
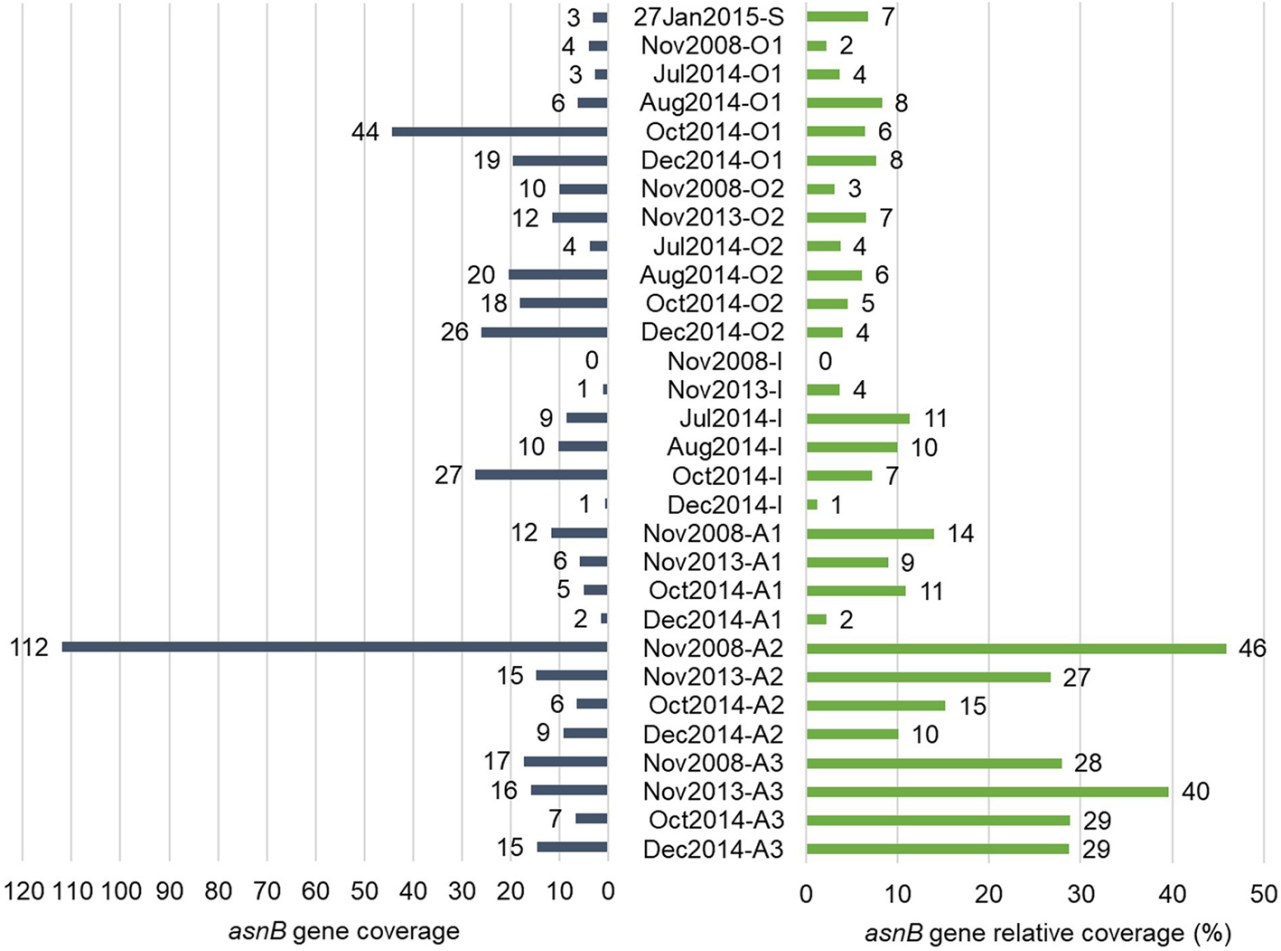
Depth profile of abundance of *Ca.* Regnicoccus frigidus *asnB* gene. Bar charts depicting glutamine-hydrolysing asparagine synthetase (*asnB*) gene coverage (left; dark blue bars) and relative coverage (right; green bars) in Ace Lake merged metagenomes. The *asnB* gene was from a *Ca.* Regnicoccus frigidus MAG generated from Dec 2014, 12 m depth, 0.8-μm filter metagenome (see ‘Methods’ for further description). Gene relative coverages (%) indicate the proportion of *Ca.* Regnicoccus frigidus population in each merged metagenome that contained the gene. The data are arranged from top to bottom by depth followed by time period of sample collection. Depths: S, surface; O1, oxic 1; O2, oxic 2; I, interface; A1, anoxic 1; A2, anoxic 2; A3, anoxic 3

Using one MAG that contained *asnB* (99.7% bin completeness, 3.6% bin contamination, Dec 2014, 12 m depth, 0.8-μm filter metagenome), significant coverage variation was found between the anoxic zone (specifically anoxic 2 and 3; average 22%) and the oxic-anoxic interface (6%) or the oxic zone (5%) (Fig. 9; Additional file 4: Dataset S3). In ammonium-rich environments, AsnB can catalyse the formation of asparagine [74], and may therefore enable the anoxic zone *Ca.* Regnicoccus frigidus population (where ammonium levels are high; Refs. [22, 69, 70]) to benefit by being able to assimilate ammonia using AsnB (Fig. 7). Conversely, in the nitrogen-limited oxic zone, by having a capacity to perform glutamine-dependent asparagine synthesis [75], the relatively small *asnB* population would be expected to have an improved capacity to utilise bioavailable nitrogen (Fig. 7). While less than half of the *Ca.* Regnicoccus frigidus population possessed *asnB*, the gene was consistently identified in metagenomes representing all lake strata (oxic, oxic-anoxic interface, anoxic) and time (2008 to 2014), indicating it was a stable feature of the population (Fig. 9).

## Conclusion

A *Synechococcus*-like species is the most abundant microorganism in the oxic zone of Ace Lake, where it blooms in response to available sunlight; it also persists in the oxic zone during long periods when sunlight is absent, as well as throughout the dark, anoxic depths of the lake [23–25]. Here, we have shown that a single *Synechococcus*-like species, composed of two major phylotypes (one more abundant than the other), colonises Ace Lake, and that the population composition varies with lake depth (Fig. 3; Additional file 1: Tables S3, S4, S6). The new species *Ca.* Regnicoccus frigidus differs (≤ 98% 16S rRNA identity) to all other characterized *Synechococcus* and *Synechococcus*-like species, including others from lakes in the neighbouring region. Members of the *Synechococcus* collective inhabit diverse aquatic and terrestrial habitats in Antarctica yet are rare members of the surrounding marine environment. Clearly, temperature alone is not an overriding factor that controls the ability of *Synechococcus* to colonise; in fact, the extent and diversity of Antarctic habitat that supports growth of *Synechococcus* testifies to the cyanobacteria having a capacity to adapt ‘happily’ to the Antarctic realm. In Ace Lake, the very abundant, phototrophic ‘neighbour’ of *Ca.* Regnicoccus frigidus, *Ca.* Chlorobium antarcticum has evolved a remarkably coherent population structure that is conserved across lake and stratified marine-basin ecosystems [29]. In contrast, based on 16S rRNA divergence, members of the *Synechococcus* collective are characterized by more variability between aquatic systems, and based on analyses of MAGs, they are characterized as having a higher extent of phylotype diversification (at least within Ace Lake). The capacity to rigorously interrogate population structure is predicated on having metagenome datasets that are of sufficient quality and size to generate MAGs and perform comparative analyses of specific species in order to characterise individual taxa and monitor variation (temporal, depth and so forth). What these studies of *Ca.* Chlorobium antarcticum and *Ca.* Regnicoccus frigidus reveal is the existence of distinct genomic/adaptive characteristics, exemplifying the ways in which microbial lineages can and do evolve to otherwise ‘common’ (Antarctic) environmental conditions.

Notable characteristics of *Ca.* Regnicoccus frigidus population included depth-related genomic variation: abundance of the main phylotypes MAG-AL1 and MAG-AL2 and extent and nature of VCRs, SNPs and indels of each of these phylotypes (Figs. 3 and 5; Additional file 1: Table S4; Additional file 4: Dataset S3). Some types of genomic variability were characterized as being relatively dynamic (e.g. temporal change in SNPs and indels) and others relatively stable (e.g. VCRs). The specific genomic variation of the phylotypes (gene differences and representation in the population) described functional variation, in particular, molecular traits ascribing interactions of *Ca.* Regnicoccus frigidus with its complement of viruses and metabolic distinctions denoting ecotypes (Fig. 7).

Viruses have been speculated to drive evolution of marine cyanobacteria leading to the development of virus-susceptible and virus-resistant host populations and enabling co-existence of hosts and viruses [60, 76, 77]. Viruses play particularly important roles in Antarctic aquatic systems, in part due to the low abundance of larger trophic predators [27]. Similar to the interactions of viruses with marine cyanobacteria, complex host-virus interactions have been described for a number of Antarctic microbially dominated systems [41, 78–86], including for Ace Lake [24, 25, 29]. The current study provides specific data about the cell surface structures and defence systems that are likely to be important in evading or neutralising viruses in order for *Ca.* Regnicoccus frigidus to grow successfully and persist throughout the water column of Ace Lake (Fig. 7; Additional file 4: Dataset S3; Additional file 7: Dataset S6).

The existence of *Ca.* Regnicoccus frigidus ecotypes with differing capacities for nitrogen utilisation (cyanide assimilation and glutamine-hydrolysing asparagine synthesis) could be rationalised within the context of available nitrogen in the lake. In Ace Lake, the overall atmospheric nitrogen level decreases with lake depth, being absent in anoxic waters below 18 m depth [69]. *Ca.* Regnicoccus frigidus cannot fix atmospheric nitrogen. However, it can or is predicted to utilise a variety of nitrogen sources including nitrate, nitrite, ammonia, cyanate, urea, peptides and amino acids [23, 25]. Bioavailable nitrogen is limiting in the oxic zone, but the anoxic zone is replete in ammonia and amino acids [22, 69, 70]. The Nit1C gene cluster in the abundant *Ca.* Regnicoccus frigidus phylotype in the oxic zone (MAG-AL1; Fig. 3b) is inferred to confer an ability to utilise free cyanide and nitriles as additional nitrogen sources, thereby improving its competitiveness in the oxic zone (Figs. 7 and 8). Utilising a different strategy, the *Ca.* Regnicoccus frigidus ecotype population containing *asnB* was more prevalent in the anoxic zone (Fig. 9), which is inferred to augment the ability of the population to assimilate ammonia as a source of available nitrogen (Fig. 7). AsnB has been experimentally characterized as preferring glutamine as substrate over ammonia but in ammonia-rich environments to utilise exogenous ammonia as well [74]. Genomic reconstruction greatly advances knowledge of genomic potential and provides a focus for specific genomic characteristics ‘of interest’. Here, we nominate *asnB* as a gene worthy of experimental evaluation. While not a trivial undertaking, as *Ca.* Regnicoccus frigidus has proven to be amenable to laboratory cultivation (SynAce01; Ref. [23]), the opportunity exists to attempt to experimentally characterise the enzyme and the cellular growth properties of the microorganism.

## Methods

### Sample collection, DNA sequencing and MAG generation

Sampling, DNA extraction, sequencing, assembly and annotation of 120 time-series Ace Lake metagenomes from 2006/2007, 2008/2009 and 2013–2015 have been described previously [25, 28, 38] (Additional file 1: Table S1). Metagenome samples were obtained in summer (Jan, Dec, Feb), winter (Jul, Aug) and spring (Oct, Nov) from seven lake depths: surface, 0 m; oxic 1, 5 m; oxic 2, 12–13 m; interface, 13–15 m; anoxic 1, 14–16 m; anoxic 2, 18–19 m; and anoxic 3, 23–24 m (Fig. 1a; Additional file 1: Table S1). The specific oxic-anoxic interface depths (13–15 m) and the anoxic depths (14–16 m) vary depending on seasonal and temporal changes in the lake water level arising from the net balance between inputs and outputs [22, 87, 88]. *Ca.* Regnicoccus frigidus MAGs were generated from the metagenomes (only one MAG per metagenome) by the IMG pipeline. For MIMAG (minimum information about MAGs; Ref. [89]) data, the MAG contig statistics N50, L50 and maximum contig length were calculated using BBMap v38.51 [90]; all other MAG quality and metadata were taken from IMG (Additional file 2: Dataset S1).

### *Ca.* Regnicoccus frigidus genomic variation

Reads from 60 metagenomes in which *Synechococcus*-like OTUs were identified were used for FR analyses (Additional file 1: Tables S1 and S4). Ace Lake 2006 metagenomes were not included due to differences in the sequencing technology and possible dataset size bias compared to the Ace Lake 2008 and 2013–2015 metagenomes [29]. Metagenome reads from 0.8–3 and 3–20 μm filter fractions from each time period and depth were pooled to create 30 merged metagenomes using a previously described method [29]. As the relative abundance of *Synechococcus*-like OTU was ≤ 0.3% in all 0.1–0.8-μm filter fraction metagenomes [25], they were excluded from FR analyses. Anoxic zone metagenomes from winter were not available due to sampling logistical constraints [25].

For the analysis of genomic variation in the *Ca.* Regnicoccus frigidus population, one MAG from the oxic zone and one MAG from the anoxic zone were selected: MAG-AL1 (IMG Bin ID: 3300023237_10), oxic zone, Jul 2014, 5 m depth, 3–20-μm filter fraction, 99.7% bin completeness and lowest bin contamination (0.09%); MAG-AL2 (IMG Bin ID: 3300023253_6), anoxic zone, Dec 2014, 14 m depth, 3–20-μm filter, 16S rRNA gene sequence different to MAG-AL1, high genome completeness (97%), and low bin contamination (0.63%). Contig and scaffold arrangements of the two *Ca.* Regnicoccus frigidus MAGs that would best represent draft genomes were determined using previously described methods [29]. Nucleotide sequences from MAG-AL1, MAG-AL2 and SynAce01 were used for manual rearrangement of contigs (Additional file 1: Table S2).

FR of metagenome reads to the two MAGs and calculation of base coverages were performed as described previously [29]. SNPs were detected from FR output BAM files using Samtools v1.10 variant calling commands bcftools mpileup and bcftools call [91, 92], with the –max-depth option adjusted to highest read depth observed in each metagenome. Variant call output files were further scanned using an in-house python script to identify SNPs with ≥ 90% frequency (i.e. at least 90% of the aligned reads contained the SNP) and insertion/deletion of multiple bases (indels). Only SNPs and indels with read depth ≥ 20 were considered. Additionally, FR of reads to a *Ca.* Regnicoccus frigidus MAG from Dec 2014, 12 m depth and 0.8-μm filter metagenome (IMG bin ID: 3300023227_3) was performed to assess the coverage variation of glutamine-hydrolysing asparagine synthetase (*asnB*), alkaline phosphatase (*pglZ*) and ATP-dependent Lon protease (*brxL*) genes in Ace Lake merged metagenomes.

Differences in gene orders of MAGs were assessed by aligning the *Ca.* Regnicoccus frigidus MAGs of ≥ 97% genome completeness to MAG-AL1 and MAG-AL2 using the blastn module of BLAST + v2.11.0 [93]. Alignments were manually parsed to identify MAG contigs with no matches, low identity matches (< 80%), low alignment fraction matches (< 50% contig length aligned) or short length matches (< 1 kb alignment length).

### GC content vs read depth analysis

GC content-read depth analysis was performed as described previously for Haloarchaea [39] and *Ca.* Chlorobium antarcticum [29]. Metagenome contigs of ≥ 10 kb length and 30–80% GC content, and *Ca.* Regnicoccus frigidus MAG contigs, were plotted using Python v3.6.4. The taxonomies of the metagenome contig clusters that were close to the *Ca.* Regnicoccus frigidus MAG contig cluster (i.e. metagenome contigs with 45–80% GC content) were determined from the IMG phylodist file-based contig taxonomies described previously [25]. These metagenome contigs were also aligned to *Ca.* Regnicoccus frigidus MAGs and the SynAce01 genome using blastn module of BLAST + v2.9.0. Alignment files and taxonomies were manually parsed, and contigs with low identity (< 95%) and high alignment fraction (> 50%) matches were further assessed to identify other cyanobacteria in Ace Lake that might be distantly related to *Ca.* Regnicoccus frigidus.

### *Ca.* Regnicoccus frigidus phylotype abundance

Median read depth of a *Ca.* Regnicoccus frigidus MAG in a merged metagenome, calculated as median of read depth values of each base in a MAG, was used to represent *Ca.* Regnicoccus frigidus population abundance in the metagenome. The abundance of a *Ca.* Regnicoccus frigidus gene in a merged metagenome was calculated as gene coverage using the formula as follows:

$$\frac{\sum_{(Gene)}Read\;depth\;of\;base}{{Total\;number\;of\;bases}_{(Gene)}}$$

where the numerator indicates the sum of the read depths of the bases in the gene, in each merged metagenome, and the denominator indicates the total number of bases in the gene.

To assess the proportion of *Ca.* Regnicoccus frigidus population containing specific variable coverage genes, the gene relative coverages were calculated using the formula as follows:

$$\frac{Gene\;coverage}{{Median\;read\;depth}_{(MAG)}}\times100$$

where *MAG* is MAG-AL1 or MAG-AL2. The numerator is the coverage of a gene from a MAG, and the denominator indicates the median read depth of the MAG in a merged metagenome. For estimation of relative coverages of *asnB*, *pglZ* and *brxL* (complete sequence) genes, the median read depth was used for the MAG from which the genes were taken (see above in ‘*Ca.* Regnicoccus frigidus genomic variation’). Average of relative coverages of gene(s) from a depth zone (oxic, oxic-anoxic interface, anoxic) was calculated by taking the mean of relative coverages of gene(s) in merged metagenomes from all depths and time periods in the zone.

### ANI, AAI, 16S rRNA gene and phylogenetic analyses

The pairwise ANI of *Ca.* Regnicoccus frigidus MAGs were performed using previously described methods [29]. AAI between MAG-AL1 and MAG-AL2 was estimated using AAI calculator online service [94, 95]. Furthermore, dDDH values and confidence intervals were calculated at the Type (Strain) Genome Server [96, 97] using the recommended settings of the Genome-to-Genome Distance Calculator 3.0 [98, 99]. SNPs were identified in the 16S rRNA genes of MAG-AL1 and MAG-AL2 using the FR data in the Integrative Genomics Viewer [100]. Of these, two SNPs at positions 217 and 231 were observed in both MAGs and in SynAce01 16S rRNAs in all merged metagenomes and were used as SNP markers. The frequencies of these SNPs, with read depths ≥ 20, were used to calculate the percentage contributions of the two MAGs to the overall *Ca.* Regnicoccus frigidus population in a merged metagenome (Fig. 3; Additional file 1: Table S4). Of the SNP frequencies at positions 217 and 231 in a MAG 16S rRNA gene in a merged metagenome, the smaller value was selected to reflect the percentage contribution of the *Ca.* Regnicoccus frigidus phylotype. For example, in oxic 2, Nov 2008 metagenome, the SNP frequencies at positions 217 (T) and 231 (T) of MAG-AL1 (normal bases: 217 A plus 231 G) were 7 and 4%, respectively (Additional file 1: Table S3). Here, the minimum value (4%) was selected to capture the percentage of reads that likely contained SNPs at both positions, and so the relative contribution of MAG-AL2 (normal bases: 217 T plus 231 T) was estimated as 4% in this metagenome (Additional file 1: Table S4). Furthermore, the read depths of the two MAGs were calculated in each metagenome by multiplying their percentage contribution and median read depth in a merged metagenome. Stringent FR of reads (with 100% identity) to 16S rRNAs from MAG-AL1, MAG-AL2 and SynAce01 was performed to evaluate if MAG-AL1 and MAG-AL2 represented distinct phylotypes. The 16S rRNA copy number of the two MAGs was evaluated by calculating their read-depth ratios, i.e. 16S rRNA SNPs median read depth divided by corresponding MAG read depth in each merged metagenome, and assessing their flanking gene annotations. The MAG read-depth ratios were compared to *Ca*. Chlorobium antarcticum 16S rRNA read-depth ratios as references, with ratios > 1 suggesting the presence of multiple copies. Global representation of *Ca.* Regnicoccus frigidus was assessed by blastn of 16S rRNA genes from MAG-AL1 and MAG-AL2 against 16S rRNA gene databases from IMG public-assembled metagenomes and public isolates (both databases accessed on 22 March 2022), using previously described methods [29]. The taxonomic novelty of *Ca.* Regnicoccus frigidus was assessed through 16S rRNA gene and whole proteome phylogeny, along with ANI and AAI analyses. Closely related MAGs and genomes were selected based on the placement of the MAGs in the GTDB reference tree using GTDB-tk v2.1.0 with database R207_v2 [16, 101]. These closely related genomes, along with proposed cyanobacterial type strains [5], were utilised for AAI analysis using CompareM v0.1.2 [102] and ANI analysis using fastANI v1.32 [103]. Pairwise comparisons of the whole genome, whole proteome and 16S rRNA gene sequences were calculated using the Genome BLAST Distance Phylogeny approach and distance formula d5 [98], as implemented at the Type (Strain) Genome Server. The resulting distances were used with FastME 2.1.6.1 [104] to infer balanced minimum evolution trees, and 100 pseudo-bootstrap replicates were performed. Based on the observed taxonomic placement, a novel species name was proposed according to the SeqCode regulations [105]. Genomes of the two *Ca*. Regnicoccus frigidus phylotypes were submitted to GenBank (accession IDs: MAG-AL1, JAOANE000000000; MAG-AL2, JAOANF000000000), and the type MAG MAG-AL1 was registered with SeqCode.

### Statistical analyses

The significance of the differences in gene coverages from different depths (oxic vs oxic-anoxic interface vs anoxic) and seasons (summer vs winter vs spring) was assessed for MAG-AL1 and MAG-AL2 using the DESeq2 R package [106] on all MAG genes. For season comparison, the samples from various time periods were grouped as summer: Dec and Jan; winter: Jul and Aug; and spring: Oct and Nov. For depth comparison, the samples from different lake depths were grouped as oxic: surface, oxic 1 and 2; oxic-anoxic interface: interface; anoxic: anoxic 1, 2 and 3. The parameters used for assessing significance from DESeq2 output were the same as described previously [29]. Genes with significant variations were considered as variable coverage genes, and their function was verified using previously described methods [29]. Read depths of MAG-AL1 and MAG-AL2 were used to assess the relationship between the *Ca.* Regnicoccus frigidus phylotype abundances and the physicochemical characteristics of Ace Lake such as depth, salinity, DO and temperature [25] (Additional file 1: Table S4). The DO values measured using a YSI Sonde in 2008 and a TOA WQC in 2013 and 2014 were normalised, as described previously [25]. Lake temperature and DO values were not available for certain time periods (Jul 2014 and Jan 2015); therefore, *Ca.* Regnicoccus frigidus phylotype abundance data were taken only from merged metagenomes for which all environmental data were available. Spearman’s rank correlation coefficients (*ρ*) were manually calculated between lake characteristics (depth, salinity, DO or temperature) and *Ca.* Regnicoccus frigidus MAG (MAG-AL1 or MAG-AL2) read depths. For this, the MAG read depths and lake characteristics were first ranked individually, and then a Pearson product moment correlation coefficient was calculated from the rank values. Statistical significance of the correlation was calculated using ANOVA (analysis of variance) regression analysis.

### *Ca.* Regnicoccus frigidus viruses

A list of viral contigs potentially associated with *Ca.* Regnicoccus frigidus was prepared by the following: (i) searching the IMG/VR v3 database (accessed on 28 June 2021) [107] to identify Ace Lake viral contigs with *Synechococcus* as their predicted host, (ii) including viral contigs in the Antarctic virus catalogue [44] that matched a cyanophage assembled from an Ace Lake metagenome (Additional file 4: Table S4 from Ref. [25]) and (iii) aligning the Antarctic virus catalogue contigs to the contigs from all *Ca.* Regnicoccus frigidus MAGs (Additional file 2: Dataset S1) to identify viral clusters or singletons with sequence similarity to *Ca.* Regnicoccus frigidus host genomes (Additional file 6: Dataset S5). Antarctic virus catalogue contigs from Ace Lake are hosted on IMG in the public scaffold set ‘Antarctic_Virus_catalogue_2020_Ace_lake’. Alignment was performed using the blastn module of BLAST + v2.11.0, and only viral contigs with > 95% identity matches were considered for further analysis. The cluster or singleton assignments of the *Ca.* Regnicoccus frigidus viruses were gathered from IMG/VR v3 database and the Antarctic virus catalogue. Additionally, putative prophage regions in MAG-AL1 and MAG-AL2 were identified by aligning MAG contigs to potential *Ca.* Regnicoccus frigidus viral contigs (determined from the three approaches described above) and two SynAce01 prophage sequences (phiSynAce1 and phiSynAce2; Ref. [30]) using the blastn module of BLAST + v2.11.0. Only contig regions with > 95% identity matches to multiple viral sequences were considered as putative prophage sequences in the two *Ca.* Regnicoccus frigidus MAGs.

### *Ca.* Regnicoccus frigidus defence genes

IMG gene annotations of MAG-AL1 and MAG-AL2 were manually parsed to identify auto-annotated defence genes associated with RM system, DISARM, BREX system and ABI mechanism (including a retron homologue). The functions of these defence genes were verified through manual annotation, as described previously [29]. The presence/absence of additional BREX system genes (*brxD*, *brxE*, *brxF*, *brxHI*, *brxHII*, *brxL*, *brxP*, *pglW*, *pglX*, *pglXI*, *pglY*, *pglZ*), DISARM genes (*drmA*, *drmB*, *drmC*, *drmD*, *drmE*) and ABI mechanism genes (*toxI*, *toxN*, *abiEi*, *abiEii*, *rnlA*, *rnlB*) in *Ca.* Regnicoccus frigidus MAGs were determined through matches of MAG proteins to reference proteins taken from previous publications (BREX genes from Ref. [56]) or NCBI. The Blastp module of DIAMOND v0.9.31 [108] was used for alignment, and only alignments with *e*-value < 10^−5^, protein identity > 30% and MAG protein coverage > 50% were considered.

## Supplementary Information

**Additional file 1:** **Supplemental data and findings about Ca. Regnicoccus frigidus.** **Supplementary text.** MAG-AL1, MAG-AL2 and SynAce01 16S rRNA gene analyses. Stable mutations in Ca. Regnicoccus frigidus phylotype genes. Seasonal variation in Ca. Regnicoccus frigidus gene coverages. Regnicoccus diversity in Ace Lake. Phase variation of Ca. Regnicoccus frigidus pglX gene. Supplementary figures: **Fig. S1.** Comparison of 16S rRNA genes, ANI, AAI and dDDH of Ace Lake Synechococcus like species phylotypes. **Fig. S2.** 16S rRNA read depth ratios of Ace Lake Synechococcus-like species phylotypes. **Fig. S3.** Read depths of 16S rRNA genes and SNP markers from Synechococcus-like species phylotypes in Ace Lake. **Fig. S4.** Alignment showing nucleotide identity between MAG-AL1, MAG-AL2 and SynAce01. **Fig. S5.** GC content vs read depth plots. **Fig. S6.** BREX and retron gene organization in Ca. Regnicoccus frigidus. **Fig. S7.** Ribosomal RNA gene organisation in Ca. Regnicoccus frigidus MAGs. Supplementary tables: **Table S1.** Ace Lake metagenomes analysed. **Table S2.** MAG-AL1 and MAG-AL2 contigs. **Table S3.** Distribution of SNPs in the 16S rRNA genes of MAG-AL1 and MAG-AL2. **Table S4.** Ace Lake merged metagenomes and physicochemical data used for genomic variation analyses of Ca. Regnicoccus frigidus MAGs. **Table S5.** Potential Ca. Regnicoccus frigidus contigs from Ace Lake metagenomes identified through GC-read depth analysis. **Table S6.** Description of Ca. Regnicoccus frigidus metabolic capacity and metadata.

**Additional file 2:** **Dataset S1.** MIMAG data for Ace Lake Synechococcus-like species.

**Additional file 3:** **Dataset S2.** Ace Lake Synechococcus-like species 16S rRNA gene alignment, ANI and AAI to reference genomes from IMG and GTDB.

**Additional file 4:** **Dataset S3.** Genomic variations in Ca. Regnicoccus frigidus MAGs.

**Additional file 5:** **Dataset S4.** Ca. Regnicoccus frigidus MAGs gene order.

**Additional file 6:** **Dataset S5.** Ca. Regnicoccus frigidus viruses and prophage regions.

**Additional file 7:** **Dataset S6.** Ca. Regnicoccus frigidus defence genes.

## Data Availability

All metagenomes and medium- and high-quality MAGs used in this study are available in IMG: see details in Additional file 1: Tables S1 and S2 and Additional file 2: Dataset S1. The draft genome of MAG-AL1, the type MAG of *Ca.* Regnicoccus frigidus, is available in GenBank (JAOANE000000000) and registered with SeqCode.
