# Supplementary material for: Population structure of an Antarctic aquatic cyanobacterium
Source: Microbiome. 2022 Dec 2;10:207. doi: 10.1186/s40168-022-01404-x (PMC9716671; doi:10.1186/s40168-022-01404-x)
Supplement: Supplementary file 2 — Additional file 1: Supplemental data and findings about Ca. Regnicoccus frigidus. Supplementary text. MAG-AL1, MAG-AL2 and SynAce01 16S rRNA gene analyses. Stable mutations in Ca. Regnicoccus frigidus phylotype genes. Seasonal variation in Ca. Regnicoccus frigidus gene coverages. Regnicoccus diversity in Ace Lake. Phase variation of Ca. Regnicoccus frigidus pglX gene. Supplementary figures: Fig. S1. Comparison of 16S rRNA genes, ANI, AAI and dDDH of Ace Lake Synechococcus like species phylotypes. Fig. S2. 16S rRNA read depth ratios of Ace Lake Synechococcus-like species phylotypes. Fig. S3. Read depths of 16S rRNA genes and SNP markers from Synechococcus-like species phylotypes in Ace Lake. Fig. S4. Alignment showing nucleotide identity between MAG-AL1, MAG-AL2 and SynAce01. Fig. S5. GC content vs read depth plots. Fig. S6. BREX and retron gene organization in Ca. Regnicoccus frigidus. Fig. S7. Ribosomal RNA gene organisation in Ca. Regnicoccus frigidus MAGs. Supplementary tables: Table S1. Ace Lake metagenomes analysed. Table S2. MAG-AL1 and MAG-AL2 contigs. Table S3. Distribution of SNPs in the 16S rRNA genes of MAG-AL1 and MAG-AL2. Table S4. Ace Lake merged metagenomes and physicochemical data used for genomic variation analyses of Ca. Regnicoccus frigidus MAGs. Table S5. Potential Ca. Regnicoccus frigidus contigs from Ace Lake metagenomes identified through GC-read depth analysis. Table S6. Description of Ca. Regnicoccus frigidus metabolic capacity and metadata. [file 40168_2022_1404_MOESM1_ESM.pdf]

# Population structure of an Antarctic aquatic cyanobacterium

Pratibha Panwar, Timothy J. Williams, Michelle A. Allen and Ricardo Cavicchioli

## Additional file 1: Supplemental data and findings about *Ca.* Regnicoccus frigidus

**Supplementary text**

MAG-AL1, MAG-AL2 and SynAce01 16S rRNA gene analyses
Stable mutations in *Ca*. Regnicoccus frigidus phylotype genes
Seasonal variation in *Ca*. Regnicoccus frigidus gene coverages
*Regnicoccus* diversity in Ace Lake
Phase variation of *Ca*. Regnicoccus frigidus *pglX* gene

**Supplementary figures**

**Fig. S1** Comparison of 16S rRNA genes, ANI, AAI and dDDH of Ace Lake *Synechococcus*-like species phylotypes.
**Fig. S2** 16S rRNA read depth ratios of Ace Lake *Synechococcus*-like species phylotypes.
**Fig. S3** Read depths of 16S rRNA genes and SNP markers from *Synechococcus*-like species phylotypes in Ace Lake.
**Fig. S4** Alignment showing nucleotide identity between MAG-AL1, MAG-AL2 and SynAce01.
**Fig. S5** GC content *vs* read depth plots.
**Fig. S6** BREX and retron gene organization in *Ca*. Regnicoccus frigidus.
**Fig. S7** Ribosomal RNA gene organisation in *Ca*. Regnicoccus frigidus MAGs.

**Supplementary tables**

**Table S1** Ace Lake metagenomes analysed.
**Table S2** MAG-AL1 and MAG-AL2 contigs.
**Table S3** Distribution of SNPs in the 16S rRNA genes of MAG-AL1 and MAG-AL2.
**Table S4** Ace Lake merged metagenomes and physicochemical data used for genomic variation analyses of *Ca*. Regnicoccus frigidus MAGs.
**Table S5** Potential *Ca*. Regnicoccus frigidus contigs from Ace Lake metagenomes identified through GC-read depth analysis.
**Table S6** Description of *Ca.* Regnicoccus frigidus metabolic capacity and metadata.

**References**

**MAG-AL1, MAG-AL2 and SynAce01 16S rRNA gene analyses**

The 16S rRNA genes identified in MAG-AL1, MAG-AL2 and SynAce01 were nearly identical (> 99.8%), with only two SNPs at positions 231 and 271: MAG-AL1 had 217 A plus 231 G; MAG-AL2 had 217 T plus 231 T; and SynAce01 had two 16S rRNA genes, 'gene 1' with 217 T plus 231 G and 'gene 2' with 217 T plus a base missing at 231 (Fig. 2; Additional file 1: Fig. S1a). The frequencies of these SNP markers (217 A-T plus 231 G-T) were used to calculate the contributions of MAG-AL1 and MAG-AL2 to the overall Ace Lake *Synechococcus*-like species population (Fig. 3; Additional file 1: Table S4). To assess whether these 16S rRNA sequences represented genes from distinct phylotypes, a stringent FR (100% identity) of reads to the 16S rRNA sequences was performed and the coverages of the SNP markers were assessed (Additional file 1: Fig. S3).

(i) *Synechococcus-like species phylotypes*. The 16S rRNA read depths and SNP marker read depths of the two MAGs were indicative of MAG-AL1 and MAG-AL2 representing two distinct *Synechococcus*-like species phylotypes (Additional file 1: Fig. S3, Table S3).

(ii) *MAG-AL1 is the most prominent Synechococcus-like species phylotype in Ace Lake*. The peak read depths of MAG-AL1 SNP markers were high, approaching 1300 in some oxic zone metagenomes, indicating this phylotype was more abundant in the oxic depths of Ace Lake than in the oxic-anoxic interface or anoxic depths (Fig. 3; Additional file 1: Fig. S3a, b, Table S4).

(iii) *MAG-AL2 is a low abundance Synechococcus-like species phylotype*. The read depths of MAG-AL2 SNP markers (peak read depth 164) were low compared to that of MAG-AL1 SNP markers (peak read depth 1278), and this phylotype was mainly represented in the oxic-anoxic interface and surrounding depths (Fig. 3; Additional file 1: Fig. S3c, d, Table S4).

(iv) SynAce01 appeared to be a rare *Synechococcus*-like species phylotype, based on the very low coverage of its SNP markers (Additional file 1: Fig. S3e–h). The read depths of the SNP markers in gene 1 (217 T plus 231 G; Additional file 1: Fig. S3f) did not match. Closer inspection of the reads aligned to the SNP markers of gene 1 showed that only one read from Oct 2014 Anoxic 2 depth had 100% identity to both SNP markers (i.e., the read included both 217 T and 231 G). All other reads aligned to gene 1, as well as those aligned to gene 2, matched 217 T or 231 G, but never both. Contrary to this, MAG-AL1 and MAG-AL2 SNP marker read depths were similar within each MAG (Additional file 1: Fig. S3b, d), and multiple reads with 100% identity contained either 217 A plus 231 G (from MAG-AL1) or 217 T plus 231 T (from MAG-AL2).

The stringent FR output, along with other genomic analyses, suggested that *Synechococcus*-like MAGs contained two identical copies of 16S rRNA genes:

(i) *Read depth ratio*. In the 100% identity FR analysis, the median read depths of the 16S rRNA SNPs from MAG-AL1 and MAG-AL2 were nearly twice that of the read depths of their corresponding MAGs (read depth ratios > 1; Additional file 1: Fig. S2). In comparison, the 16S rRNA read depth ratio of *Ca*. Chlorobium antarcticum, which contains only one copy of 16S rRNA gene, was ~1 in all metagenomes.

(ii) *Genes flanking 5S and 16S rRNAs*. A total of 25 *Synechococcus*-like MAGs contained two copies of 5S rRNAs and three contained additional partial sequences (≤ 101 bp) of 16S rRNAs at contig ends. The genes upstream of 16S rRNA genes, or downstream of identical 5S rRNA genes, from a MAG were different (Additional file 1: Fig. S7). For example, MAG-AL1 contained two identical 5S rRNA sequences at contig ends, but the genes downstream of these two 5S rRNAs were different (Additional file 1: Fig. S7d, e). Moreover, a combination of 16S rRNA plus its upstream genes and 5S rRNA plus its downstream genes was observed in different MAGs with identical rRNA genes (Additional

file 1: Fig. S7). These permutations could be explained by the presence of two identical rRNA gene clusters with differing flanking genes placed in different regions of each MAG. The identical rRNA genes would cause mis-assembly leading to different combinations of rRNA gene clusters and their flanking genes, which is also supported by their location at contig ends.

The two 16S rRNAs from SynAce01 demonstrated some disparities that were not observed in MAG-AL1 and MAG-AL2: the two genes were not identical and had different SNP markers (217 T plus 231 G or 217 T plus a base missing at 231; Fig. 2; Additional file 1: Fig. S1a), the read depths of gene 1 SNP markers were different (Additional file 1: Fig. S3f), and only one read from Oct 2014 Anoxic 2 depth matched both SNP markers of gene 1. These observations might be related to issues with SynAce01 16S rRNA assembly. SynAce01 genome was assembled [1] from an isolate extracted from a non-axenic and non-clonal culture [2]. It is possible that the culture contained multiple, closely-related *Synechococcus*-like strains, which would be very difficult to differentiate morphologically, as is the case with *Synechococcus* species. To assess this possibility, we performed FR analyses of the SynAce01 reads available in NCBI SRA (Illumina: SRX2338505; PacBio: SRX2347259) to SynAce01 16S rRNA genes. Notably, the reads matching both genes contained either 217 A plus 231 G (as for MAG-AL1) or 217 T plus 231 T (as for MAG-AL2), and no other combination. Similar observations were made during FR analyses of Ace Lake merged metagenome reads to SynAce01 16S rRNA genes. Further, alignment of SynAce01 genome to MAG-AL1 and MAG-AL2 contigs revealed SynAce01 regions that aligned to MAG-AL1 but not MAG-AL2 or vice versa. Together, these data indicated that SynAce01 might represent a pan-genome of Ace Lake *Synechococcus*-like species phylotypes that had grown during laboratory cultivation.

**Stable mutations in *Ca.* Regnicoccus frigidus phylotype genes**

Mutations (SNPs and indels) were observed in 45 MAG-AL1 and 157 MAG-AL2 genes but only a few of these (in two MAG-AL1 and 10 MAG-AL2 genes) were identified as stable, i.e., mutations observed in metagenomes from different time periods at a depth. The stable SNPs led to non-synonymous substitutions, whereas the stable insertions (of multiple bases) caused frame-shift mutations and insertion of additional amino acids in the protein sequence. Both types of mutations are likely to affect protein function as they altered the protein sequences, except for one MAG-AL2 gene (vitamin K epoxide reductase family protein), in which multiple bases were inserted after the stop codon that did not alter its protein sequence.

MAG-AL1 genes containing stable mutations encoded: a 2-oxoisovalerate dehydrogenase with a frame-shift mutation at protein position 47 and a 4-Amino-4-deoxy-L-arabinose transferase-like glycosyltransferase with a non-synonymous substitution (W→R) at protein position 329. MAG-AL2 genes containing stable mutations encoded: a carboxysome shell carbonic anhydrase with a non-synonymous substitution (Q→L) at protein position 18; a glycerol-3-phosphate acyltransferase PlsX with additional amino acids (AGSV) inserted at protein position 52; a N-acetylglucosamine-6-phosphate deacetylase with additional amino acids (WGGAG) inserted at protein position 388, which was not within the catalytic domain of the protein; a UDP-glucuronate decarboxylase with non-synonymous substitution (N→T) at protein position 54 in its putative NAD binding site, which might affect its cofactor binding; a glycosyltransferase family 2 protein containing WcaA domain with non-synonymous substitution (S→R) at protein position 146; a hypothetical protein with a frame-shift mutation at protein position 37; a hypothetical protein with additional amino acids (HLE) at protein position 30; a hypothetical protein with an additional amino acid (R) at protein position 27; and a hypothetical protein with non-synonymous substitution (D→E) at protein position 603 followed by additional inserted amino acids (RKKYRA).

## Seasonal variation in *Ca*. Regnicoccus frigidus gene coverages

The *Ca*. Regnicoccus frigidus MAG genes did not exhibit significant coverage variations between seasonal samples. We considered why this might be the case. *Ca*. Regnicoccus frigidus is a phototroph, and seasonal change in sunlight affects its abundance in the oxic zone [3]. Highest relative abundance was observed in summer (Dec ≤ 58%), lowest in early winter (Jul ≤ 6%) and re-establishing in late winter (Aug ≤ 16%) and spring (Oct, Nov ≤ 51%) [3]. Here, we also observed a similar pattern of median read depths of both *Ca*. Regnicoccus frigidus phylotypes (Additional file 1: Table S4); however, there was overlap between the ranges of median read depths. For example, *Ca*. Regnicoccus frigidus MAG oxic zone median read depths were 76–340 in winter (Aug 2014) which overlapped with spring (Nov 2008, 174–325; Nov 2013, 180–181) and summer (Dec 2014, 256–659). Manual assessment of the confidence intervals of the seasonal statistics revealed complete overlap between summer, winter and spring groups. Generally, non-overlapping confidence intervals necessarily indicate significant differences, but differences between statistics with overlapping confidence intervals may not necessarily be insignificant. Therefore, the lack of significant gene coverage variations during comparison of seasonal samples may not be true, and may have arisen as a result of the similarity between samples from winter *vs* summer and spring.

## *Regnicoccus* diversity in Ace Lake

Of the 51,971 metagenome contigs clustered around *Ca*. Regnicoccus frigidus MAG contigs (Additional file 1: Fig. S5), only 297 were cyanobacterial contigs. More than half of these cyanobacterial contigs (165) had ≥ 99% identity matches (across ≥ 90% of their length) to *Ca*. Regnicoccus frigidus MAG contigs; one contig had 96% identity match across its whole length and a few other contigs (13) had > 99.5% identity matches across 54–85% of their length (Additional file 1: Table S5). These contigs might belong to *Ca*. Regnicoccus frigidus MAGs in metagenomes from which either no MAGs or low bin completeness MAGs were generated, or they might represent closely-related *Ca*. Regnicoccus frigidus phylotype contigs in metagenomes from which high bin completeness MAGs were generated (Additional file 1: Table S5). One cyanobacterial contig (IMG scaffold ID: Ga0222650_1000802) from the metagenome from which MAG-AL1 was assembled, had good matches to a number of *Ca*. Regnicoccus frigidus MAG contigs, including MAG-AL2 (IMG scaffold ID: Ga0222695_1000681; ~100% identity across 80% contig length). This contig likely belonged to a different *Ca*. Regnicoccus frigidus phylotype in that metagenome. Genes on this contig encoded a retron homolog (529 bp length), TA proteins, DNA repair protein, symporter, transposases, and hypothetical and general function proteins. Remaining cyanobacterial contigs either did not align or had short length matches to *Ca*. Regnicoccus frigidus MAGs. Overall, diverse *Ca*. Regnicoccus frigidus phylotypes, other than the closely-related phylotypes identified through phylogenetic and FR analyses, were not detected in Ace Lake.

## Phase variation of *Ca*. Regnicoccus frigidus *pglX* gene

Various gene interruptions, such as gene duplication, inversion and truncation, have been reported to be a part of *pglX* gene phase variation, which is associated with change in its specificity function in BREX systems or attenuation of its probable toxicity in the absence of viral infection [4]. The *pglX* gene in *Ca*. Regnicoccus frigidus MAGs were truncated and found at or near contig ends, with some MAGs containing two truncated *pglX* genes (coding for 583–615 aa length proteins). Of the two truncated *pglX* genes, one was annotated close to *pglZ* and *brxL* genes, whereas the other was usually found adjacent to a transposase (Additional file 1: Fig. S6; Additional file 7: Dataset S6). The two truncated PglX MAG

proteins matched different regions of the reference PglX protein, and together represented a complete PglX protein (~1200 aa). In *Methanobrevibacter smithii* and two *Lactobacillus rhamnosus* GG strains, a complete *pglX* gene and a recombinase were identified adjacent to truncated *pglX* genes, and the recombinase was implicated in shuffling DNA between the complete and truncated forms of *pglX*, allowing for phase variation of the gene [4]. A similar process for *pglX* gene phase variation, using an adjacent transposase, could possibly be employed by *Ca*. Regnicoccus frigidus.

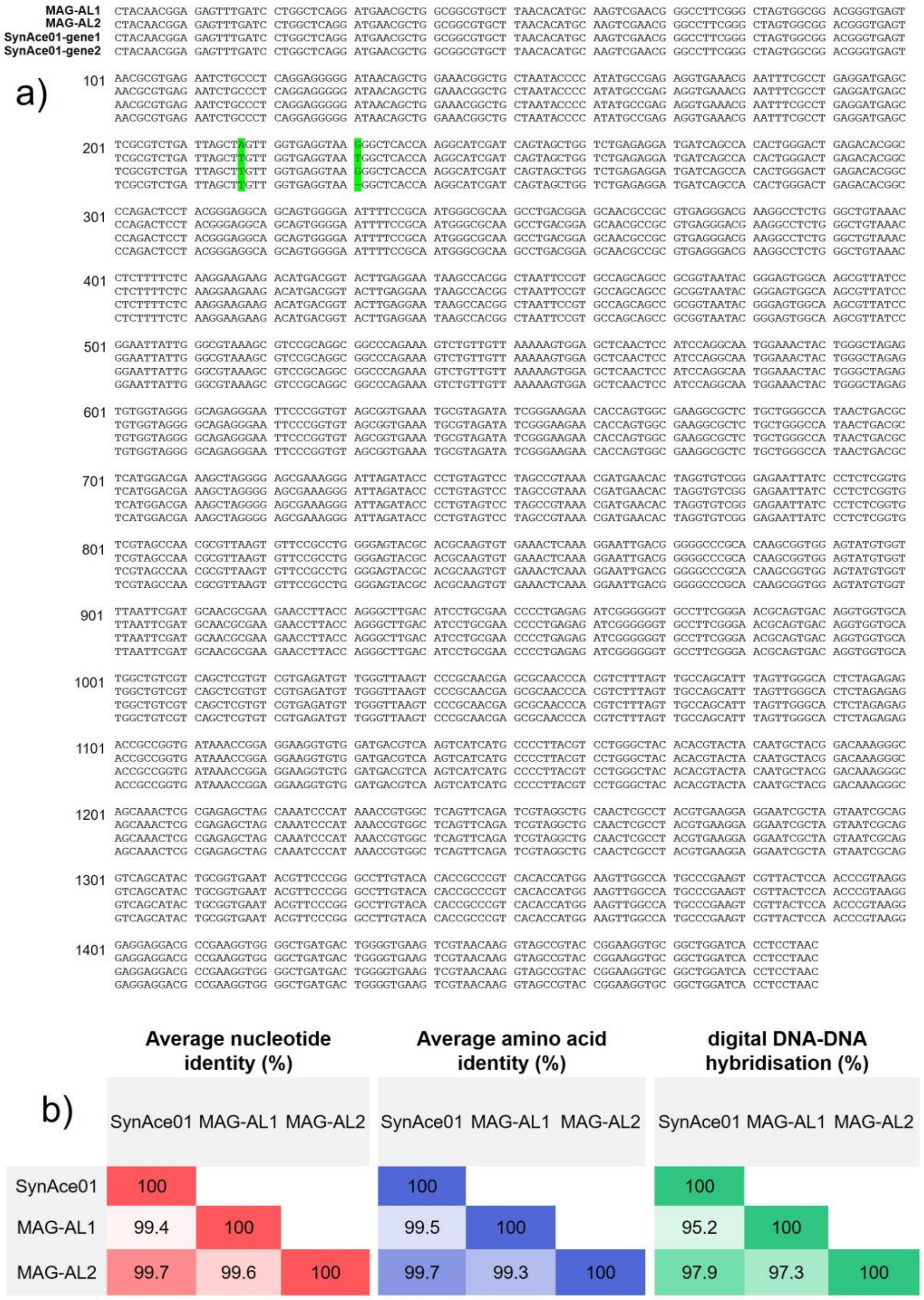


**Fig. S1** Comparison of 16S rRNA genes, ANI, AAI and dDDH of Ace Lake *Synechococcus*-like species phylotypes. (**a**) Mismatch sites at positions 217 and 231 (highlighted in green); sequence gap at position 231 of SynAce01-gene2 is represented by a ‘dash’ symbol. The 16S rRNA genes were taken from IMG: MAG-AL1, Ga0222650_100050630; MAG-AL2, Ga0222695_100009760; SynAce01-gene1, 2721490753; SynAce01-gene2, 2721489686. (**b**) Pair-wise ANI and AAI of MAG-AL1, MAG-AL2 and SynAce01 were > 99%, indicating that the three genomes were very similar. This was supported by high (> 95%) dDDH of the three genomes suggesting that they belonged to the same species and subspecies. AAI, average amino acid identity; ANI, average nucleotide identity; dDDH, digital DNA-DNA hybridisation.

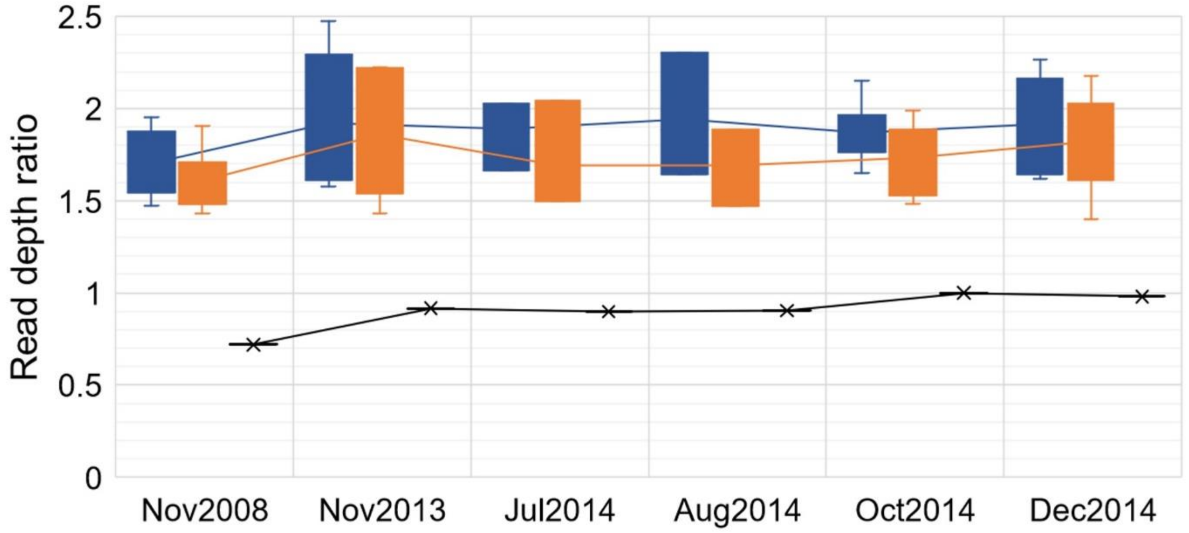


**Fig. S2** 16S rRNA read depth ratios of Ace Lake *Synechococcus*-like species phylotypes. Box plot showing the 16S rRNA read depth ratios of MAG-AL1 (blue boxes) and MAG-AL2 (orange boxes), compared to *Ca*. Chlorobium antarcticum (black line). Ratios were calculated by dividing the median read depths of 16S rRNA SNPs (Additional file 1: Fig. S3b, d) or full-length 16S rRNA gene by the read depths of the corresponding *Synechococcus*-like species phylotype or *Ca*. Chlorobium antarcticum MAG in each merged metagenome, respectively. These values were used to assess the copy number of 16S rRNA genes in MAGs, with ratio ~2 indicating presence of two copies of 16S rRNA gene in *Synechococcus*-like MAGs; consistent with SynAce01 containing two 16S rRNA genes. The x-axis shows data arranged by time period. For Ace Lake *Synechococcus*-like species phylotypes, the ratios from different depth metagenomes in a time period were grouped in the box plots, with the lines joining the mean values in each time period. For Ace Lake *Ca*. Chlorobium antarcticum, the ratios were calculated only in the oxic-anoxic interface merged metagenomes (pooled data from all three filter fractions) from each time period.

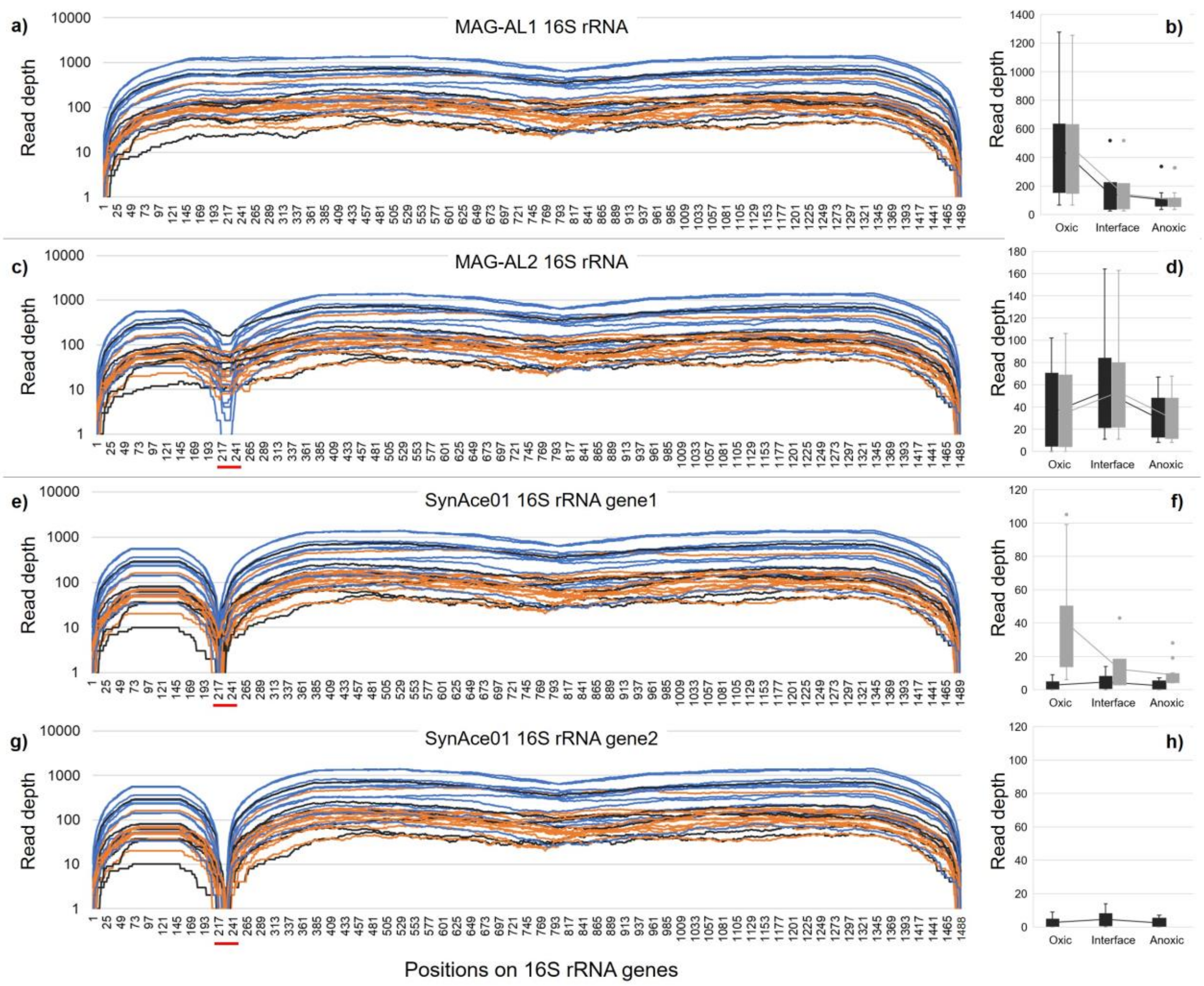


**Fig. S3** Read depths of 16S rRNA genes and SNP markers from *Synechococcus*-like species phylotypes in Ace Lake. The line graphs (**a**, **c**, **e**, **g**) depict the base coverages of 16S rRNA genes and the box plots (**b**, **d**, **f**, **h**) show the read depths of SNPs at positions 217 (black boxes) and 231 (grey boxes) of 16S rRNAs from MAG-AL1, MAG-AL2 and SynAce01. The x-axes in **a**, **c**, **e** and **g** show positions on 16S rRNA genes: red lines below axes in **c**, **e** and **g** highlight SNP regions containing SNPs at positions 217 and 231. The y-axes display read depths on a log scale (**a**, **c**, **e**, **g**) or linear scale (**b**, **d**, **f**, **h**). The read depths were generated through stringent FR (100% identity) of reads from merged metagenomes from various lake depths (blue, oxic depths; black, oxic-anoxic interface; orange, anoxic depths) to *Synechococcus*-like species 16S rRNA genes. For the box plots (**b**, **d**, **f**, **h**), the SNP read depths from different depth metagenomes (oxic, oxic-anoxic interface, anoxic) were grouped, with the lines joining the mean values in each zone. SynAce01 16S rRNA gene 2 (1,488 bp length) is missing a base at position 231 compared to the other three 16S rRNA genes (1,489 bp length; Fig. 2; Additional file 1: Fig. S1a), therefore its read depth at position 231 (**h**) is shown as zero in all metagenomes.

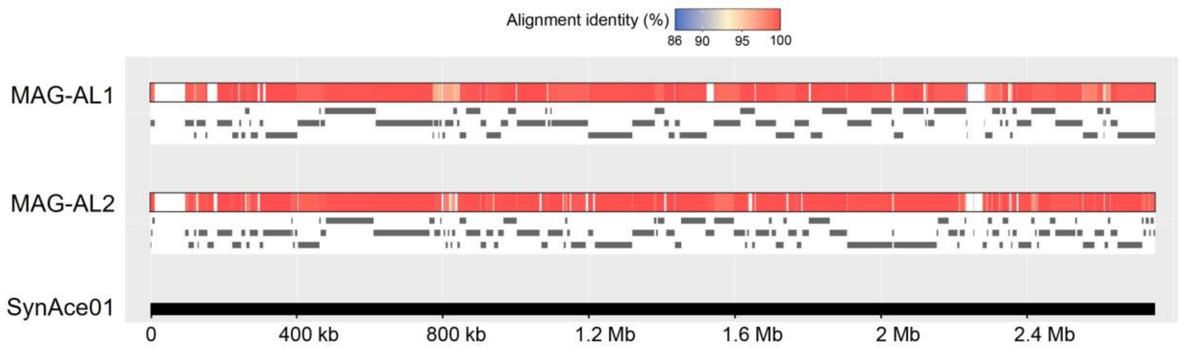


**Fig. S4** Alignment showing nucleotide identity between MAG-AL1, MAG-AL2 and SynAce01. Reference SynAce01 genome with x-axis labels denoting genome base pair positions (thick black line); nucleotide identity between MAG and reference (coloured bars); alignment gaps denoting no match between MAG and reference (white regions in bars); MAG contigs aligned to reference (grey dashes under bars), where the length of dashes represents contig alignment length (minimum length 1 kb). MAG contigs that did not align to the reference genome are not shown. The gradient bar denotes percentage nucleotide identity from 86% (blue) to 100% (red).

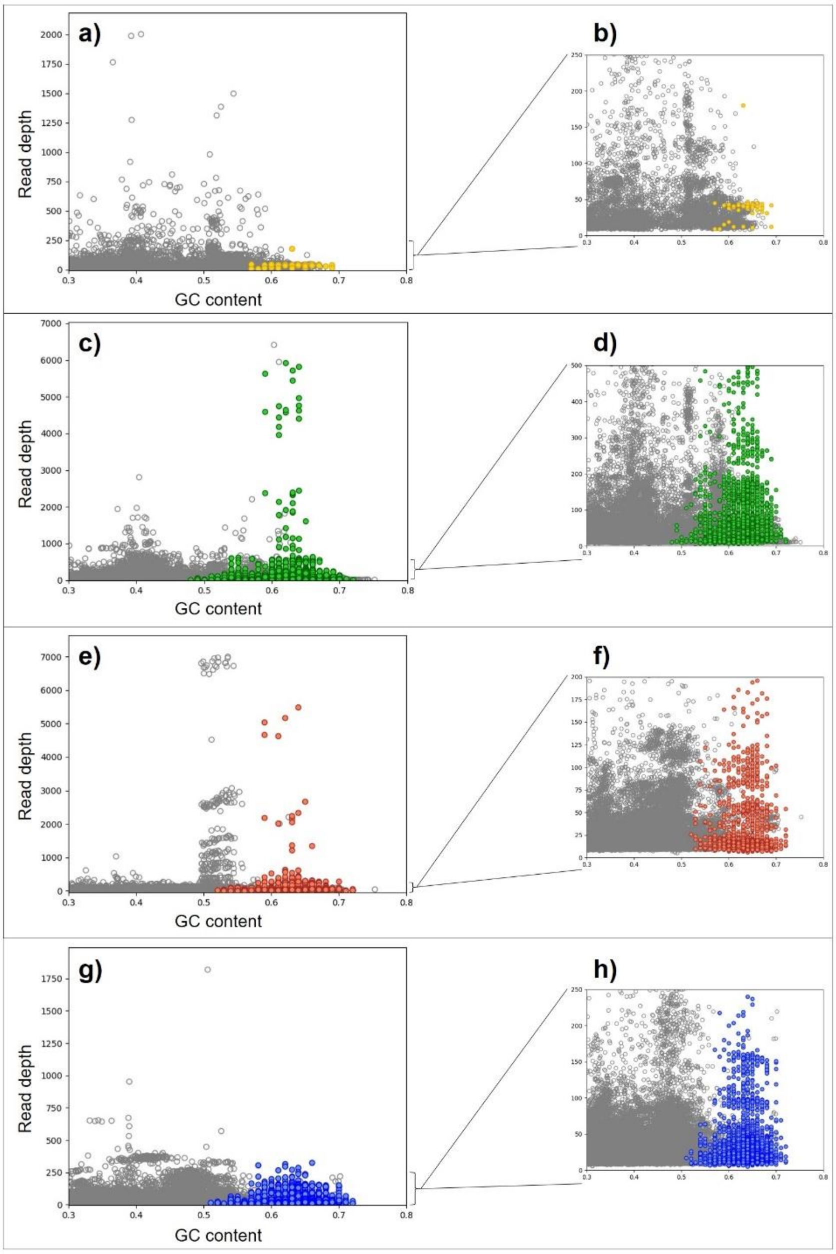


**Fig. S5** GC content *vs* read depth plots. Scatter plots showing the grouping of *Ca*. Regnicoccus frigidus MAG contigs from Ace Lake surface (**a**, **b**; yellow circles), oxic zone (**c**, **d**; green circles), oxic-anoxic interface (**e**, **f**; orange circles), and anoxic zone (**g**, **h**; blue circles) as well as metagenome contigs (grey empty circles) from the respective lake depths on a GC-read depth 2D space. Panels **b**, **d**, **f** and **h** show magnified view of plot sections to highlight the clustering of low read depth metagenome contigs.

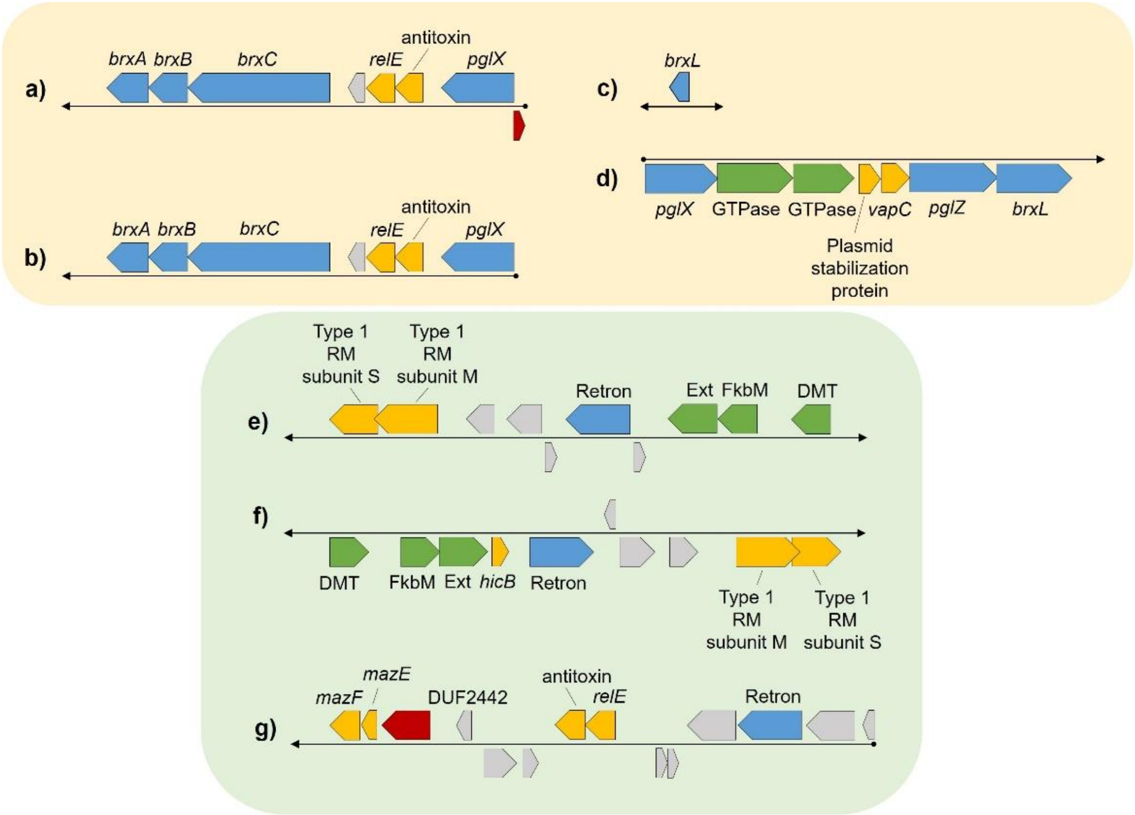


**Fig. S6** BREX and retron gene organization in *Ca*. Regnicoccus frigidus. Schematic showing the arrangement of BREX type 1 system genes (yellow background) and retron homologs (green background) in *Ca*. Regnicoccus frigidus MAG contigs: blue, BREX or retron genes; yellow, TA or RM genes; green, other metabolic genes; grey, hypothetical or unknown function genes; dark red, transposases; black horizontal line, contig backbone with black dots representing contig ends and black arrows showing contig continuity. (**a**, **b**) The gene cluster containing *brxA*, *brxB*, *brxC* and *pglX* (truncated) was observed in almost all *Ca*. Regnicoccus frigidus MAGs, with either a transposase (**a**) or *pglX* (**b**) at contig end; MAG-AL1 and MAG-AL2 contained a transposase at contig end. (**c**) A truncated *brxL* gene, flanked by other metabolic genes, was identified on the contigs of some MAGs, including MAG-AL1 and MAG-AL2. (**d**) Gene cluster containing complete sequences of *pglZ* and *brxL* genes and an additional truncated *pglX* gene were observed only in some *Ca*. Regnicoccus frigidus MAGs (Additional file 7: Dataset S6). (**e, f**) Retron homolog (607 aa length) identified close to restriction enzyme genes had a hypothetical gene downstream of it, and either an exostosin family domain-containing gene (**e**; observed in MAG-AL1) or *hicB* antitoxin gene (**f**) upstream of it. (**g**) Another retron homolog (529 aa length) was found at the ends of *Ca*. Regnicoccus frigidus MAG contigs, with TA system genes and a transposase encoded nearby. BREX, Bacteriophage Exclusion; *brxA*, BREX protein BrxA; *brxB*, BREX protein BrxB; *brxC*, BREX system P-loop protein BrxC; *brxL*, ATP-dependent Lon protease; DMT, drug/metabolite transporter-like permease; Ext, exostosin family domain-containing protein; FkbM, methyltransferase FkbM domain-containing protein; *hicB*, type II TA system HicB family antitoxin; *mazE*, type II TA system MazE family antitoxin; *mazF*, type II TA system MazF family toxin; *pglX*, adenine-specific methyltransferase; *pglZ,* alkaline phosphatase; *relE*, type II TA system RelE/ParE family toxin; RM, restriction-modification; TA, toxin-antitoxin; *vapC*, type II toxin-antitoxin system VapC family toxin.

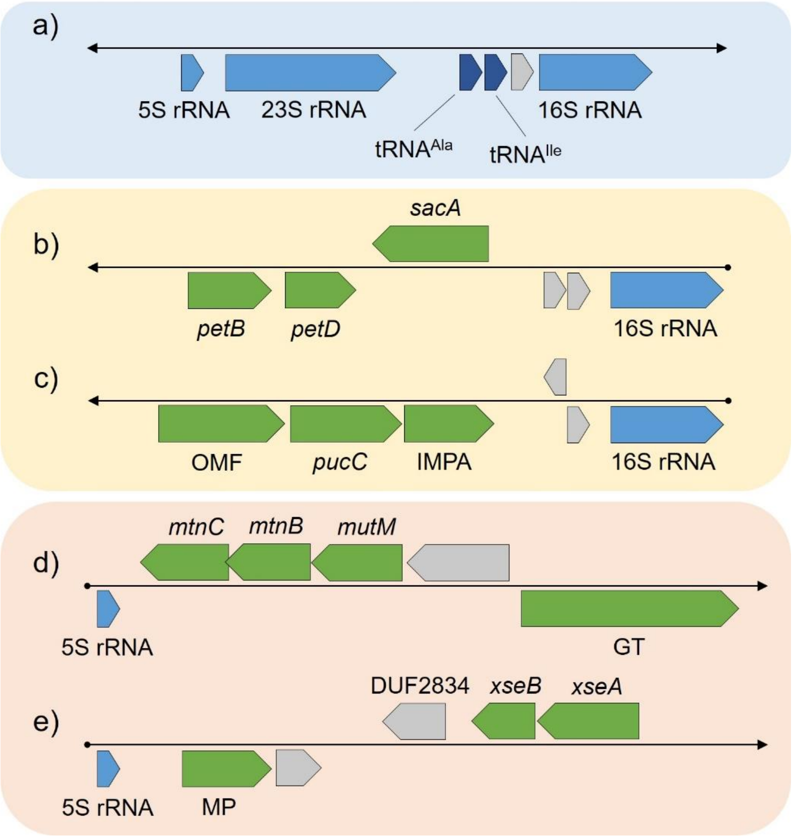


**Fig. S7** Ribosomal RNA gene organisation in *Ca*. Regnicoccus frigidus MAGs. The schematic shows the arrangement of ribosomal RNA and their flanking genes in 59 high- and medium-quality *Ca*. Regnicoccus frigidus MAGs: blue shapes, 5S, 16S, 23S rRNAs; green shapes, metabolic genes; dark blue shapes, tRNAs; grey shapes, hypothetical or uncharacterised genes; black lines, contig backbones with arrows indicating contig continuity and dots representing contig ends. (**a**) The 16S–23S intergenic spacer region of *Ca*. Regnicoccus frigidus MAGs encoded two tRNAs and a hypothetical gene, similar to SynAce01. The genes upstream of 16S rRNAs (**b**, **c**; yellow background) and downstream of 5S rRNAs (**d**, **e**; orange background) differed within MAGs containing two 16S rRNAs (the second copy being a partial sequence $\leq$ 101 bp at contig end) and between MAGs with identical 5S and 16S rRNA genes. Genes: DUF2834, uncharacterized protein DUF2834; GT, glycosyltransferase involved in cell wall biosynthesis; IMPA, myo-inositol-1(or 4)-monophosphatase; MP, membrane protein; *mtnB*, methylthioribulose-1-phosphate dehydratase; *mtnC*, enolase-phosphatase E1; *mutM*, formamidopyrimidine-DNA glycosylase; OMF, OMF family outer membrane factor; *petB*, cytochrome b6; *petD*, cytochrome b6-f complex subunit 4; *pucC*, BCD family chlorophyll transporter-like MFS transporter; *sacA*, beta-fructofuranosidase; *xseB*, exodeoxyribonuclease VII small subunit; *xseA*, exodeoxyribonuclease VII large subunit.

**Table S1** Ace Lake metagenomes analysed.

| Sample collection date (DD/MM/YYYY); depth; filter fraction | Lake depth[A] | IMG genome ID | Metagenome filtered reads (bp) | Assembled metagenome size (bp)[B] | Total protein-coding genes |
|---|---|---|---|---|---|
| 20/12/2006; 5 m; 3 µm | Oxic 1 | 3300028202 | 65,944,407 | 9,717,163 | 18,015 |
| 20/12/2006; 5 m; 0.8 µm | Oxic 1 | 3300028221 | 188,760,566 | 27,952,213 | 53,952 |
| 20/12/2006; 5 m; 0.1 µm | Oxic 1 | 3300028228 | 514,425,517 | 33,518,956 | 64,687 |
| 20/12/2006; 11.5 m; 3 µm | Oxic 2 | 3300028205 | 152,109,562 | 22,138,314 | 39,285 |
| 20/12/2006; 11.5 m; 0.8 µm | Oxic 2 | 3300028289 | 194,556,802 | 16,906,227 | 32,171 |
| 20/12/2006; 11.5 m; 0.1 µm | Oxic 2 | 3300028222 | 501,692,433 | 29,126,306 | 60,086 |
| 20/12/2006; 12.7 m; 3 µm | Interface | 3300028203 | 83,214,739 | 10,703,483 | 20,757 |
| 20/12/2006; 12.7 m; 0.8 µm | Interface | 3300028201 | 208,538,507 | 11,925,309 | 23,740 |
| 20/12/2006; 12.7 m; 0.1 µm | Interface | 3300028204 | 240,290,391 | 6,971,450 | 13,087 |
| 20/12/2006; 14 m; 3 µm | Anoxic 1 | 3300028200 | 118,655,678 | 15,468,656 | 31,907 |
| 20/12/2006; 14 m; 0.8 µm | Anoxic 1 | 3300028302 | 165,208,287 | 27,504,336 | 56,468 |
| 20/12/2006; 14 m; 0.1 µm | Anoxic 1 | 3300028219 | 169,703,894 | 23,317,396 | 54,216 |
| 20/12/2006; 18 m; 3 µm | Anoxic 2 | 3300028199 | 114,460,928 | 12,486,049 | 26,210 |
| 20/12/2006; 18 m; 0.8 µm | Anoxic 2 | 3300028227 | 214,177,665 | 34,270,862 | 71,009 |
| 20/12/2006; 18 m; 0.1 µm | Anoxic 2 | 3300028216 | 145,906,502 | 15,860,072 | 40,100 |
| 20/12/2006; 23 m; 3 µm | Anoxic 3 | 3300028292 | 105,388,116 | 11,819,279 | 24,794 |
| 20/12/2006; 23 m; 0.8 µm | Anoxic 3 | 3300028226 | 231,162,768 | 33,899,871 | 71,413 |
| 20/12/2006; 23 m; 0.1 µm | Anoxic 3 | 3300028296 | 292,220,289 | 26,024,886 | 62,208 |
| 19/11/2008; 5 m; 3 µm | Oxic 1 | 3300025601 | 10,168,447,444 | 374,845,559 | 637,417 |
| 19/11/2008; 5 m; 0.8 µm | Oxic 1 | 3300025513 | 8,608,322,293 | 358,461,005 | 555,436 |
| 19/11/2008; 5 m; 0.1 µm | Oxic 1 | 3300025425 | 9,326,252,194 | 190,824,688 | 354,920 |
| 21/11/2008; 11.8 m; 3 µm | Oxic 2 | 3300025502 | 9,958,328,840 | 309,922,874 | 529,432 |
| 21/11/2008; 11.8 m; 0.8 µm | Oxic 2 | 3300025603 | 10,372,524,015 | 387,727,814 | 649,215 |
| 21/11/2008; 11.8 m; 0.1 µm | Oxic 2 | 3300025438 | 8,652,779,583 | 208,281,887 | 381,283 |
| 21/11/2008; 12.8 m; 3 µm | Interface | 3300025433 | 7,377,945,147 | 191,332,554 | 330,516 |
| 21/11/2008; 12.8 m; 0.8 µm | Interface | 3300025380 | 7,969,400,898 | 118,925,863 | 224,047 |
| 21/11/2008; 12.8 m; 0.1 µm | Interface | 3300025362 | 15,030,492,867 | 90,472,821 | 190,960 |
| 21/11/2008; 14.1 m; 3 µm | Anoxic 1 | 3300025649 | 8,878,877,148 | 403,510,882 | 775,430 |
| 21/11/2008; 14.1 m; 0.8 µm | Anoxic 1 | 3300025628 | 9,024,438,900 | 379,168,081 | 728,210 |
| 21/11/2008; 14.1 m; 0.1 µm | Anoxic 1 | 3300025697 | 7,433,358,222 | 401,517,242 | 923,143 |
| 21/11/2008; 18 m; 3 µm | Anoxic 2 | 3300025642 | 9,701,518,914 | 444,311,389 | 775,322 |
| 21/11/2008; 18 m; 0.8 µm | Anoxic 2 | 3300025586 | 10,550,636,481 | 338,938,472 | 589,716 |
| 21/11/2008; 18 m; 0.1 µm | Anoxic 2 | 3300025669 | 8,489,799,212 | 415,535,816 | 832,930 |
| 23/11/2008; 23 m; 3 µm | Anoxic 3 | 3300025698 | 8,926,498,848 | 428,043,704 | 894,948 |
| 23/11/2008; 23 m; 0.8 µm | Anoxic 3 | 3300025661 | 8,835,913,368 | 414,688,901 | 822,281 |
| 23/11/2008; 23 m; 0.1 µm | Anoxic 3 | 3300025736 | 8,391,237,271 | 477,169,979 | 1,113,701 |
| 24/11/2013; 5 m; 3 µm | Oxic 1 | 3300022867 | 4,225,013,370 | 144,719,058 | 289,211 |
| 24/11/2013; 5 m; 0.8 µm | Oxic 1 | 3300023243 | 4,462,325,958 | 205,826,389 | 369,592 |
| 24/11/2013; 5 m; 0.1 µm | Oxic 1 | 3300022843 | 3,805,948,564 | 100,883,143 | 212,850 |
| 25/11/2013; 12.5 m; 3 µm | Oxic 2 | 3300022842 | 4,534,814,707 | 163,226,887 | 302,245 |

| | | | | | |
|---|---|---|---|---|---|
| 25/11/2013; 12.5 m; 0.8 µm | Oxic 2 | 3300022847 | 4,208,778,962 | 155,718,155 | 244,054 |
| 25/11/2013; 12.5 m; 0.1 µm | Oxic 2 | 3300023235 | 4,703,733,094 | 143,622,133 | 282,929 |
| 26/11/2013; 13.5 m; 3 µm | Interface | 3300022882 | 4,632,992,773 | 197,528,912 | 370,963 |
| 26/11/2013; 13.5 m; 0.8 µm | Interface | 3300023244 | 4,017,414,066 | 152,968,368 | 281,280 |
| 26/11/2013; 13.5 m; 0.1 µm | Interface | 3300022871 | 4,289,343,500 | 153,918,125 | 304,781 |
| 26/11/2013; 15 m; 3 µm | Anoxic 1 | 3300023234 | 2,830,397,582 | 132,062,988 | 251,704 |
| 26/11/2013; 15 m; 0.8 µm | Anoxic 1 | 3300022854 | 4,179,971,653 | 189,382,169 | 349,194 |
| 26/11/2013; 15 m; 0.1 µm | Anoxic 1 | 3300023435 | 3,982,384,098 | 204,889,614 | 458,784 |
| 26/11/2013; 19 m; 3 µm | Anoxic 2 | 3300023298 | 3,861,886,442 | 173,692,067 | 351,338 |
| 26/11/2013; 19 m; 0.8 µm | Anoxic 2 | 3300023262 | 5,356,530,473 | 256,708,329 | 493,455 |
| 26/11/2013; 19 m; 0.1 µm | Anoxic 2 | 3300023297 | 4,526,133,618 | 236,042,504 | 568,485 |
| 27/11/2013; 24 m; 3 µm | Anoxic 3 | 3300022828 | 2,032,322,733 | 65,695,823 | 149,469 |
| 27/11/2013; 24 m; 0.8 µm | Anoxic 3 | 3300022887 | 4,489,480,975 | 197,136,157 | 423,504 |
| 27/11/2013; 24 m; 0.1 µm | Anoxic 3 | 3300031227 | 21,163,513,792 | 1,050,144,399 | 2,413,590 |
| 17/12/2013; 0 m; 3 µm | Surface | 3300022841 | 3,505,709,238 | 109,878,484 | 205,134 |
| 17/12/2013; 0 m; 0.8 µm | Surface | 3300022833 | 3,007,301,388 | 112,095,376 | 172,874 |
| 17/12/2013; 0 m; 0.1 µm | Surface | 3300022822 | 3,926,440,146 | 72,848,168 | 141,301 |
| 15/02/2014; 0 m; 3 µm | Surface | 3300022827 | 4,445,471,441 | 150,261,289 | 262,344 |
| 15/02/2014; 0 m; 0.8 µm | Surface | 3300023054 | 4,101,153,533 | 186,668,359 | 262,345 |
| 15/02/2014; 0 m; 0.1 µm | Surface | 3300022839 | 4,105,154,760 | 94,401,441 | 195,630 |
| 2/07/2014; 5 m; 3 µm | Oxic 1 | 3300023237 | 4,712,346,032 | 179,194,270 | 291,313 |
| 2/07/2014; 5 m; 0.8 µm | Oxic 1 | 3300022866 | 4,450,973,256 | 227,490,836 | 403,969 |
| 2/07/2014; 5 m; 0.1 µm | Oxic 1 | 3300022853 | 4,388,723,345 | 128,153,264 | 250,568 |
| 3/07/2014; 12.5 m; 3 µm | Oxic 2 | 3300022857 | 3,349,508,936 | 162,523,775 | 274,815 |
| 3/07/2014; 12.5 m; 0.8 µm | Oxic 2 | 3300022836 | 3,812,123,689 | 173,061,625 | 297,508 |
| 3/07/2014; 12.5 m; 0.1 µm | Oxic 2 | 3300023245 | 4,389,831,560 | 141,659,134 | 285,227 |
| 3/07/2014; 13.5 m; 3 µm | Interface | 3300022834 | 3,025,335,676 | 150,334,734 | 279,053 |
| 3/07/2014; 13.5 m; 0.8 µm | Interface | 3300023241 | 3,917,460,255 | 176,108,874 | 316,827 |
| 3/07/2014; 13.5 m; 0.1 µm | Interface | 3300023257 | 4,754,144,028 | 246,566,898 | 516,984 |
| 20/08/2014; 5 m; 3 µm | Oxic 1 | 3300023236 | 3,535,315,349 | 145,971,573 | 260,745 |
| 20/08/2014; 5 m; 0.8 µm | Oxic 1 | 3300023239 | 3,675,443,392 | 161,858,999 | 300,236 |
| 20/08/2014; 5 m; 0.1 µm | Oxic 1 | 3300023229 | 3,581,244,138 | 112,903,843 | 219,283 |
| 21/08/2014; 13 m; 3 µm | Oxic 2 | 3300022885 | 4,805,699,185 | 232,800,896 | 422,661 |
| 21/08/2014; 13 m; 0.8 µm | Oxic 2 | 3300022845 | 3,046,800,658 | 127,278,965 | 240,608 |
| 21/08/2014; 13 m; 0.1 µm | Oxic 2 | 3300023296 | 4,126,784,684 | 163,100,043 | 305,555 |
| 21/08/2014; 14.5 m; 3 µm | Interface | 3300022864 | 4,208,293,249 | 203,541,480 | 379,585 |
| 21/08/2014; 14.5 m; 0.8 µm | Interface | 3300024048 | 4,438,778,032 | 185,710,747 | 327,952 |
| 21/08/2014; 14.5 m; 0.1 µm | Interface | 3300022890 | 3,761,803,592 | 196,439,047 | 427,804 |
| 20/10/2014; 5 m; 3 µm | Oxic 1 | 3300022865 | 3,718,130,970 | 159,691,784 | 283,171 |
| 20/10/2014; 5 m; 0.8 µm | Oxic 1 | 3300022825 | 3,500,964,757 | 137,992,510 | 261,144 |
| 20/10/2014; 5 m; 0.1 µm | Oxic 1 | 3300023294 | 4,051,255,334 | 135,330,843 | 259,473 |
| 20/10/2014; 12 m; 3 µm | Oxic 2 | 3300022848 | 3,461,486,260 | 157,234,838 | 316,382 |
| 20/10/2014; 12 m; 0.8 µm | Oxic 2 | 3300023238 | 3,185,298,810 | 140,908,866 | 262,229 |
| 20/10/2014; 12 m; 0.1 µm | Oxic 2 | 3300023240 | 3,685,976,302 | 125,847,023 | 262,910 |
| 21/10/2014; 13 m; 3 µm | Interface | 3300022856 | 3,793,702,914 | 185,885,369 | 366,842 |
| 21/10/2014; 13 m; 0.8 µm | Interface | 3300022859 | 3,615,901,126 | 148,572,713 | 281,988 |

| | | | | | |
|---|---|---|---|---|---|
| 21/10/2014; 13 m; 0.1 µm | Interface | 3300022821 | 3,169,765,298 | 119,795,036 | 247,086 |
| 21/10/2014; 16 m; 3 µm | Anoxic 1 | 3300022855 | 2,823,639,110 | 137,224,766 | 262,841 |
| 21/10/2014; 16 m; 0.8 µm | Anoxic 1 | 3300023249 | 3,472,734,434 | 161,447,324 | 294,441 |
| 21/10/2014; 16 m; 0.1 µm | Anoxic 1 | 3300022858 | 3,214,387,734 | 162,887,351 | 368,840 |
| 21/10/2014; 19 m; 3 µm | Anoxic 2 | 3300023434 | 3,699,374,508 | 165,008,949 | 330,503 |
| 21/10/2014; 19 m; 0.8 µm | Anoxic 2 | 3300022838 | 3,195,707,102 | 158,062,637 | 299,108 |
| 21/10/2014; 19 m; 0.1 µm | Anoxic 2 | 3300023246 | 3,202,188,919 | 153,939,570 | 372,354 |
| 21/10/2014; 24 m; 3 µm | Anoxic 3 | 3300023251 | 3,707,575,608 | 149,036,067 | 306,831 |
| 21/10/2014; 24 m; 0.8 µm | Anoxic 3 | 3300023295 | 4,015,996,994 | 166,137,713 | 367,296 |
| 21/10/2014; 24 m; 0.1 µm | Anoxic 3 | 3300022874 | 3,523,521,042 | 181,923,112 | 450,383 |
| 4/12/2014; 5 m; 3 µm | Oxic 1 | 3300023501 | 3,558,906,481 | 126,636,802 | 250,738 |
| 4/12/2014; 5 m; 0.8 µm | Oxic 1 | 3300022844 | 3,528,199,602 | 163,618,968 | 306,086 |
| 4/12/2014; 5 m; 0.1 µm | Oxic 1 | 3300023293 | 3,287,944,538 | 81,894,154 | 178,097 |
| 4/12/2014; 12 m; 3 µm | Oxic 2 | 3300023231 | 3,372,774,996 | 116,441,688 | 240,321 |
| 4/12/2014; 12 m; 0.8 µm | Oxic 2 | 3300023227 | 3,766,666,990 | 103,396,553 | 207,492 |
| 4/12/2014; 12 m; 0.1 µm | Oxic 2 | 3300022851 | 3,582,064,538 | 119,299,278 | 248,470 |
| 4/12/2014; 13.4 m; 3 µm | Interface | 3300031697 | 14,149,086,706 | 400,324,806 | 718,959 |
| 4/12/2014; 13.4 m; 0.8 µm | Interface | 3300022826 | 2,989,229,242 | 78,299,135 | 145,800 |
| 4/12/2014; 13.4 m; 0.1 µm | Interface | 3300023292 | 3,878,932,484 | 85,111,111 | 181,733 |
| 4/12/2014; 14 m; 3 µm | Anoxic 1 | 3300023253 | 3,420,681,173 | 167,955,693 | 307,470 |
| 4/12/2014; 14 m; 0.8 µm | Anoxic 1 | 3300023233 | 3,250,064,514 | 144,877,168 | 252,928 |
| 4/12/2014; 14 m; 0.1 µm | Anoxic 1 | 3300022868 | 3,895,509,417 | 195,190,896 | 414,173 |
| 3/12/2014; 19 m; 3 µm | Anoxic 2 | 3300022860 | 4,079,964,767 | 181,977,179 | 369,802 |
| 3/12/2014; 19 m; 0.8 µm | Anoxic 2 | 3300022846 | 3,983,828,178 | 165,102,958 | 309,999 |
| 3/12/2014; 19 m; 0.1 µm | Anoxic 2 | 3300023061 | 3,209,269,596 | 152,256,002 | 384,107 |
| 3/12/2014; 24 m; 3 µm | Anoxic 3 | 3300022884 | 4,021,442,672 | 179,261,304 | 381,611 |
| 3/12/2014; 24 m; 0.8 µm | Anoxic 3 | 3300023299 | 5,006,350,890 | 217,304,898 | 440,798 |
| 3/12/2014; 24 m; 0.1 µm | Anoxic 3 | 3300023256 | 3,621,396,862 | 179,844,837 | 445,634 |
| 8/01/2015; 0 m; 3 µm | Surface | 3300022829 | 3,645,848,765 | 78,301,103 | 152,629 |
| 8/01/2015; 0 m; 0.8 µm | Surface | 3300022832 | 3,757,499,746 | 136,667,441 | 270,106 |
| 8/01/2015; 0 m; 0.1 µm | Surface | 3300023242 | 3,407,544,904 | 121,628,756 | 269,881 |
| 27/01/2015; 0 m; 3 µm | Surface | 3300023230 | 3,829,689,694 | 116,684,467 | 219,301 |
| 27/01/2015; 0 m; 0.8 µm | Surface | 3300023429 | 3,298,326,784 | 165,138,532 | 262,012 |
| 27/01/2015; 0 m; 0.1 µm | Surface | 3300022837 | 3,616,258,196 | 93,765,159 | 194,928 |

[A] Lake depths were named depending on which Ace Lake zone they referred to: Surface, surface waters; Oxic 1 and 2, oxic zone depths; Interface, oxic-anoxic interface; Anoxic 1, 2 and 3, anoxic zone depths. [B] Assembled metagenome size is the total length of all contigs assembled from a metagenome. The orange-highlighted metagenomes were pooled to create merged metagenomes (Additional file 1: Table S4) and used to analyse *Synechococcus*-like species genomic variation. All metagenomes were used for analysis of GC-read depth and *Synechococcus*-like species defence genes, and viral contigs from all metagenomes were used for *Synechococcus*-like species virus analysis. Filter fractions: 3, 3–20 µm; 0.8, 0.8–3 µm; 0.1, 0.1–0.8 µm.

**Table S2** MAG-AL1 and MAG-AL2 contigs.

| **Contig number**[A] | **IMG scaffold ID** | **Length (bp)** | **GC content** | **Read depth**[B] |
|---|---|---|---|---|
| MAG-AL1 (IMG bin ID: 3300023237_10) | | | | |
| 1 | Ga0222650_1000034 | 137,101 | 0.64 | 21 |
| 2 | Ga0222650_1002821 | 6,356 | 0.65 | 18 |
| 3 | Ga0222650_1000619 | 25,620 | 0.61 | 18 |
| 4 | Ga0222650_1004114 | 4,742 | 0.63 | 15 |
| 5 | Ga0222650_1000312 | 40,490 | 0.6 | 17 |
| 6 | Ga0222650_1001062 | 16,055 | 0.62 | 16 |
| 7 | Ga0222650_1003914 | 4,925 | 0.6 | 17 |
| 8 | Ga0222650_1001677 | 10,260 | 0.6 | 18 |
| 9 | Ga0222650_1001440 | 11,806 | 0.62 | 24 |
| 10 | Ga0222650_1005579 | 3,734 | 0.65 | 16 |
| 11 | Ga0222650_1000914 | 18,649 | 0.61 | 18 |
| 12 | Ga0222650_1001868 | 9,303 | 0.57 | 21 |
| 13 | Ga0222650_1000081 | 86,813 | 0.64 | 19 |
| 14 | Ga0222650_1000319 | 39,964 | 0.63 | 21 |
| 15 | Ga0222650_1000026 | 167,373 | 0.65 | 20 |
| 16 | Ga0222650_1000522 | 28,922 | 0.58 | 22 |
| 17 | Ga0222650_1001396 | 12,177 | 0.65 | 18 |
| 18 | Ga0222650_1000170 | 59,315 | 0.65 | 22 |
| 19 | Ga0222650_1001100 | 15,498 | 0.65 | 22 |
| 20 | Ga0222650_1000001 | 295,825 | 0.66 | 22 |
| 21 | Ga0222650_1000923 | 18,473 | 0.68 | 21 |
| 22 | Ga0222650_1001967 | 8,836 | 0.6 | 20 |
| 23 | Ga0222650_1000129 | 67,306 | 0.65 | 20 |
| 24 | Ga0222650_1000467 | 31,382 | 0.63 | 20 |
| 25 | Ga0222650_1000506 | 29,683 | 0.65 | 22 |
| 26 | Ga0222650_1001026 | 16,650 | 0.65 | 21 |
| 27 | Ga0222650_1000984 | 17,396 | 0.68 | 21 |
| 28 | Ga0222650_1000209 | 53,520 | 0.64 | 22 |
| 29 | Ga0222650_1000331 | 39,404 | 0.66 | 21 |
| 30 | Ga0222650_1000857 | 19,483 | 0.64 | 19 |
| 31 | Ga0222650_1000761 | 21,785 | 0.63 | 18 |
| 32 | Ga0222650_1000087 | 82,453 | 0.6 | 20 |
| 33 | Ga0222650_1001945 | 8,931 | 0.65 | 18 |
| 34 | Ga0222650_1002673 | 6,655 | 0.67 | 19 |
| 35 | Ga0222650_1005344 | 3,861 | 0.68 | 15 |
| 36 | Ga0222650_1000063 | 99,630 | 0.65 | 21 |
| 37 | Ga0222650_1000043 | 120,828 | 0.65 | 22 |
| 38 | Ga0222650_1000594 | 26,364 | 0.67 | 20 |
| 39 | Ga0222650_1000158 | 61,308 | 0.64 | 23 |
| 40 | Ga0222650_1000197 | 55,170 | 0.64 | 21 |

| | | | | |
|---|---|---|---|---|
| 41 | Ga0222650_1000222 | 50,320 | 0.64 | 20 |
| 42 | Ga0222650_1000338 | 38,996 | 0.62 | 20 |
| 43 | Ga0222650_1000132 | 66,151 | 0.64 | 22 |
| 44 | Ga0222650_1000194 | 55,721 | 0.65 | 20 |
| 45 | Ga0222650_1000459 | 31,919 | 0.63 | 19 |
| 46 | Ga0222650_1000119 | 71,760 | 0.61 | 19 |
| 47 | Ga0222650_1003846 | 4,990 | 0.6 | 15 |
| 48 | Ga0222650_1004646 | 4,295 | 0.7 | 15 |
| 49 | Ga0222650_1001012 | 16,953 | 0.67 | 20 |
| 50 | Ga0222650_1000069 | 94,031 | 0.66 | 21 |
| 51 | Ga0222650_1000773 | 21,576 | 0.65 | 20 |
| 52 | Ga0222650_1000778 | 21,461 | 0.65 | 21 |
| 53 | Ga0222650_1002879 | 6,263 | 0.68 | 18 |
| 54 | Ga0222650_1001854 | 9,357 | 0.68 | 19 |
| 55 | Ga0222650_1002499 | 7,021 | 0.63 | 20 |
| 56 | Ga0222650_1000638 | 25,222 | 0.66 | 20 |
| 57 | Ga0222650_1000008 | 219,105 | 0.64 | 21 |
| 58 | Ga0222650_1001081 | 15,714 | 0.64 | 21 |
| 59 | Ga0222650_1005666 | 3,703 | 0.58 | 19 |
| 60 | Ga0222650_1000413 | 34,181 | 0.64 | 21 |
| 61 | Ga0222650_1000762 | 21,780 | 0.63 | 89 |
| 62 | Ga0222650_1005153 | 3,975 | 0.57 | 23 |
| 63 | Ga0222650_1001237 | 13,690 | 0.61 | 18 |
| 64 | Ga0222650_1000749 | 22,092 | 0.66 | 19 |
| MAG-AL2 (IMG bin ID: 3300023253_6) | | | | |
| 1 | Ga0222695_1000064 | 67,194 | 0.64 | 22 |
| 2 | Ga0222695_1003498 | 4,759 | 0.64 | 18 |
| 3 | Ga0222695_1002402 | 6,387 | 0.62 | 21 |
| 4 | Ga0222695_1001795 | 8,069 | 0.62 | 24 |
| 5 | Ga0222695_1004560 | 3,861 | 0.63 | 19 |
| 6 | Ga0222695_1001841 | 7,935 | 0.66 | 19 |
| 7 | Ga0222695_1002743 | 5,752 | 0.64 | 19 |
| 8 | Ga0222695_1004782 | 3,716 | 0.69 | 16 |
| 9 | Ga0222695_1000760 | 15,491 | 0.66 | 20 |
| 10 | Ga0222695_1000863 | 14,169 | 0.63 | 22 |
| 11 | Ga0222695_1002754 | 5,731 | 0.65 | 21 |
| 12 | Ga0222695_1005879 | 3,138 | 0.62 | 16 |
| 13 | Ga0222695_1000425 | 23,484 | 0.6 | 21 |
| 14 | Ga0222695_1000184 | 40,497 | 0.6 | 21 |
| 15 | Ga0222695_1000417 | 23,821 | 0.62 | 21 |
| 16 | Ga0222695_1001318 | 10,270 | 0.6 | 21 |
| 17 | Ga0222695_1001334 | 10,146 | 0.64 | 22 |
| 18 | Ga0222695_1000478 | 21,660 | 0.62 | 21 |
| 19 | Ga0222695_1001482 | 9,323 | 0.57 | 23 |

| | | | | |
|---|---|---|---|---|
| 20 | Ga0222695_1000048 | 77,323 | 0.64 | 21 |
| 21 | Ga0222695_1001467 | 9,397 | 0.63 | 15 |
| 22 | Ga0222695_1004569 | 3,852 | 0.61 | 17 |
| 23 | Ga0222695_1004016 | 4,265 | 0.6 | 13 |
| 24 | Ga0222695_1002423 | 6,354 | 0.57 | 27 |
| 25 | Ga0222695_1002057 | 7,230 | 0.61 | 23 |
| 26 | Ga0222695_1003589 | 4,661 | 0.67 | 16 |
| 27 | Ga0222695_1002905 | 5,513 | 0.62 | 21 |
| 28 | Ga0222695_1000293 | 30,588 | 0.65 | 22 |
| 29 | Ga0222695_1000114 | 53,502 | 0.65 | 21 |
| 30 | Ga0222695_1000422 | 23,654 | 0.65 | 19 |
| 31 | Ga0222695_1000066 | 67,014 | 0.66 | 21 |
| 32 | Ga0222695_1000189 | 40,103 | 0.6 | 21 |
| 33 | Ga0222695_1000077 | 63,356 | 0.65 | 21 |
| 34 | Ga0222695_1000586 | 18,510 | 0.65 | 23 |
| 35 | Ga0222695_1000014 | 130,319 | 0.66 | 22 |
| 36 | Ga0222695_1000009 | 153,028 | 0.66 | 21 |
| 37 | Ga0222695_1000876 | 13,956 | 0.69 | 19 |
| 38 | Ga0222695_1000713 | 16,024 | 0.65 | 19 |
| 39 | Ga0222695_1005412 | 3,363 | 0.64 | 20 |
| 40 | Ga0222695_1000141 | 46,908 | 0.66 | 21 |
| 41 | Ga0222695_1000097 | 57,387 | 0.62 | 25 |
| 42 | Ga0222695_1004269 | 4,067 | 0.64 | 20 |
| 43 | Ga0222695_1003036 | 5,312 | 0.67 | 20 |
| 44 | Ga0222695_1004630 | 3,805 | 0.63 | 18 |
| 45 | Ga0222695_1004346 | 4,010 | 0.69 | 18 |
| 46 | Ga0222695_1001789 | 8,093 | 0.67 | 19 |
| 47 | Ga0222695_1002607 | 5,980 | 0.67 | 21 |
| 48 | Ga0222695_1000621 | 17,926 | 0.67 | 21 |
| 49 | Ga0222695_1000113 | 53,540 | 0.64 | 22 |
| 50 | Ga0222695_1000598 | 18,263 | 0.66 | 21 |
| 51 | Ga0222695_1001150 | 11,287 | 0.66 | 19 |
| 52 | Ga0222695_1000772 | 15,340 | 0.64 | 21 |
| 53 | Ga0222695_1000234 | 35,998 | 0.64 | 21 |
| 54 | Ga0222695_1000080 | 61,902 | 0.61 | 21 |
| 55 | Ga0222695_1000685 | 16,570 | 0.58 | 17 |
| 56 | Ga0222695_1000180 | 40,763 | 0.65 | 21 |
| 57 | Ga0222695_1002978 | 5,387 | 0.61 | 18 |
| 58 | Ga0222695_1002663 | 5,884 | 0.64 | 19 |
| 59 | Ga0222695_1002112 | 7,048 | 0.69 | 16 |
| 60 | Ga0222695_1000193 | 39,664 | 0.65 | 21 |
| 61 | Ga0222695_1000924 | 13,512 | 0.66 | 20 |
| 62 | Ga0222695_1000034 | 101,648 | 0.65 | 21 |
| 63 | Ga0222695_1003669 | 4,586 | 0.69 | 15 |

| | | | | |
|---|---|---|---|---|
| 64 | Ga0222695_1000626 | 17,756 | 0.68 | 19 |
| 65 | Ga0222695_1002481 | 6,226 | 0.62 | 18 |
| 66 | Ga0222695_1000090 | 59,476 | 0.65 | 23 |
| 67 | Ga0222695_1000228 | 36,365 | 0.64 | 22 |
| 68 | Ga0222695_1001980 | 7,470 | 0.66 | 21 |
| 69 | Ga0222695_1001116 | 11,578 | 0.63 | 24 |
| 70 | Ga0222695_1000512 | 20,717 | 0.66 | 21 |
| 71 | Ga0222695_1001667 | 8,538 | 0.65 | 19 |
| 72 | Ga0222695_1000441 | 22,943 | 0.6 | 25 |
| 73 | Ga0222695_1001053 | 12,201 | 0.6 | 22 |
| 74 | Ga0222695_1000641 | 17,488 | 0.65 | 20 |
| 75 | Ga0222695_1000017 | 123,194 | 0.65 | 22 |
| 76 | Ga0222695_1000019 | 121,783 | 0.63 | 21 |
| 77 | Ga0222695_1004550 | 3,867 | 0.67 | 19 |
| 78 | Ga0222695_1000324 | 28,645 | 0.65 | 22 |
| 79 | Ga0222695_1000403 | 24,391 | 0.67 | 21 |
| 80 | Ga0222695_1003086 | 5,253 | 0.67 | 15 |
| 81 | Ga0222695_1000973 | 12,931 | 0.63 | 21 |
| 82 | Ga0222695_1002807 | 5,661 | 0.65 | 15 |
| 83 | Ga0222695_1001751 | 8,243 | 0.63 | 16 |
| 84 | Ga0222695_1001209 | 10,939 | 0.63 | 19 |
| 85 | Ga0222695_1005964 | 3,101 | 0.66 | 18 |
| 86 | Ga0222695_1001263 | 10,623 | 0.67 | 19 |
| 87 | Ga0222695_1000605 | 18,232 | 0.66 | 20 |
| 88 | Ga0222695_1001633 | 8,667 | 0.68 | 17 |
| 89 | Ga0222695_1000806 | 14,733 | 0.66 | 20 |
| 90 | Ga0222695_1005944 | 3,108 | 0.65 | 13 |
| 91 | Ga0222695_1004523 | 3,886 | 0.68 | 13 |
| 92 | Ga0222695_1000745 | 15,630 | 0.65 | 18 |
| 93 | Ga0222695_1000160 | 43,779 | 0.66 | 19 |
| 94 | Ga0222695_1004487 | 3,907 | 0.65 | 17 |
| 95 | Ga0222695_1003613 | 4,643 | 0.56 | 28 |
| 96 | Ga0222695_1000297 | 30,463 | 0.64 | 21 |
| 97 | Ga0222695_1000060 | 69,524 | 0.65 | 21 |
| 98 | Ga0222695_1003806 | 4,454 | 0.6 | 30 |
| 99 | Ga0222695_1000148 | 45,489 | 0.65 | 21 |
| 100 | Ga0222695_1000382 | 25,519 | 0.65 | 21 |
| 101 | Ga0222695_1000394 | 24,659 | 0.64 | 21 |
| 102 | Ga0222695_1001605 | 8,801 | 0.62 | 21 |
| 103 | Ga0222695_1001885 | 7,758 | 0.65 | 124 |
| 104 | Ga0222695_1001830 | 7,971 | 0.62 | 56 |
| 105 | Ga0222695_1000681 | 16,583 | 0.56 | 22 |
| 106 | Ga0222695_1002505 | 6,195 | 0.61 | 99 |
| 107 | Ga0222695_1005886 | 3,133 | 0.55 | 24 |

| | | | | |
|---|---|---|---|---|
| 108 | Ga0222695_1001031 | 12,415 | 0.51 | 17 |
| 109 | Ga0222695_1004505 | 3,896 | 0.61 | 10 |
| 110 | Ga0222695_1000980 | 12,909 | 0.55 | 16 |
| 111 | Ga0222695_1000406 | 24,250 | 0.59 | 180 |
| 112 | Ga0222695_1000974 | 12,924 | 0.6 | 176 |
| 113 | Ga0222695_1001988 | 7,452 | 0.58 | 218 |
| 114 | Ga0222695_1002096 | 7,095 | 0.64 | 193 |
| 115 | Ga0222695_1002380 | 6,415 | 0.62 | 189 |
| 116 | Ga0222695_1005195 | 3,485 | 0.63 | 183 |
| 117 | Ga0222695_1003955 | 4,327 | 0.63 | 8 |
| 118 | Ga0222695_1005701 | 3,216 | 0.62 | 8 |
| 119 | Ga0222695_1005391 | 3,375 | 0.56 | 11 |
| 120 | Ga0222695_1004896 | 3,651 | 0.57 | 11 |

MAG-AL1 and MAG-AL2 are high-quality *Synechococcus*-like MAGs with > 99% and ~97% genome completeness, respectively. The two MAGs were generated from Ace Lake metagenomes from the oxic zone (Jul 2014, 5 m depth, 3–20 μm filter) and anoxic zone (Dec 2014, 14 m depth, 3–20 μm filter), respectively. [A] MAG-AL1 contigs 1–64 and MAG-AL2 contigs 1–120 correspond to contigs in Fig. 5. Scaffold arrangement of MAG contigs is shown using a common background colour, and scaffold sequences that match between the two MAGs are shown using the same background colour. MAG-AL1 contigs 63 and 64 and MAG-AL2 contigs 105–120 did not match between the two MAGs. [B] Read depths of MAG contigs are from the metagenomes in IMG that the MAGs were generated from.

**Table S3** Distribution of SNPs in the 16S rRNA genes of MAG-AL1 and MAG-AL2.

| ***Synechococcus*-like MAG**[A] | | | **MAG-AL1** | | **MAG-AL2** | | **MAG-AL1 and MAG-AL2** | | | | | | |
|---|---|---|---|---|---|---|---|---|---|---|---|---|---|
| Position on MAG 16S rRNA gene | | | 217 | 231 | 217 | 231 | 236 | 245 | 246 | 262 | 348 | 815 | 820 |
| Nucleotide on MAG | | | A | G | T | T | C | A | T | C | C | T | T |
| SNP | | | T | T | A | G | T | G | A | T | A | A | A |
| SNP frequency (%) in merged metagenomes from different lake depths and time periods[B] | Surface | Jan 2015 | 18 | 1 | 99 | 76 | 21–23 | 21–24 | 22–25 | 21–24 | 24 | | |
| | Oxic 1 | Nov 2008 | 1 | 1 | 99 | 99 | | | | | | | |
| | | Jul 2014 | 13 | 5 | 94 | 87 | | | | | | | |
| | | Aug 2014 | 18 | 4 | 96 | 80 | 16–17 | 17–19 | 17–19 | 17–18 | 17–18 | | |
| | | Oct 2014 | 2 | 2 | 98 | 98 | | | | | | | |
| | | Dec 2014 | 2 | 2 | 98 | 98 | | | | | | | |
| | Oxic 2 | Nov 2008 | 7 | 4 | 96 | 92 | 3–4 | 4–5 | 4 | 4–5 | 4–5 | 5 | 3 |
| | | Nov 2013 | 25 | 24 | 75 | 74 | | | | | | | |
| | | Jul 2014 | 7 | 6 | 94 | 93 | | | | | | | |
| | | Aug 2014 | 17 | 16 | 83 | 82 | | | | | | | |
| | | Oct 2014 | 9 | 9 | 91 | 90 | | | | | | | |
| | | Dec 2014 | 9 | 8 | 91 | 91 | | | | | | | |
| | Interface | Nov 2008 | 32 | 29 | 68 | 68 | | | | | | | |
| | | Nov 2013 | 38 | 37 | 61 | 60 | | | | | | | |
| | | Jul 2014 | 25 | 24 | 75 | 74 | | | | | | | |
| | | Aug 2014 | 30 | 30 | 68 | 68 | | | | | | | |
| | | Oct 2014 | 24 | 24 | 75 | 75 | | | | | | | |
| | | Dec 2014 | 49 | 49 | 51 | 51 | | | | | | | |
| | Anoxic 1 | Nov 2008 | 19 | 18 | 80 | 80 | | | | | | | |
| | | Nov 2013 | 35 | 33 | 66 | 64 | | | | | | | |
| | | Oct 2014 | 22 | 22 | 78 | 78 | | | | | | | |
| | | Dec 2014 | 48 | 49 | 49 | 49 | | | | | | | |
| | Anoxic 2 | Nov 2008 | 14 | 14 | 85 | 85 | | | | | | | |
| | | Nov 2013 | 29 | 26 | 73 | 70 | | | | | | | |
| | | Oct 2014 | 24 | 25 | 75 | 71 | | | | | | | |
| | | Dec 2014 | 22 | 23 | 75 | 77 | | | | | | | |

| | | | | | | | |
|---|---|---|---|---|---|---|---|
| | Anoxic 3 | Nov 2008 | 22 | 19 | 80 | 76 | |
| | | Nov 2013 | 17 | 15 | 85 | 83 | |
| | | Oct 2014 | 18 | 18 | 80 | 79 | |
| | | Dec 2014 | 13 | 14 | 86 | 87 | |

[A] *Synechococcus*-like MAG(s) containing the marker gene in which the SNPs were observed. [B] Merged metagenomes are arranged by lake depth followed by time period (see Additional file 1: Table S4 for depth description). Only SNPs with read depth ≥ 20 were considered during analysis in the Integrative Genomics Viewer (see "Methods" for description). For comparison between MAG-AL1 and MAG-AL2 16S rRNA genes, the SNP frequencies at positions 217 and 231 with read depth < 20 are shown in red font.

**Table S4** Ace Lake merged metagenomes and physicochemical data used for genomic variation analyses of *Ca*. Regnicoccus frigidus MAGs.

| Lake depth | Sample collection depth | Sample collection time period | Merged metagenome reads[A] | *Synechococcus*-like OTU relative abundance (%)[B] | | *Ca.* Regnicoccus frigidus MAG median (mean) read depth[C] | | Phylotype contribution to overall *Ca.* Regnicoccus frigidus population (%)[D] | | Ace Lake physicochemical data[E] | | |
|---|---|---|---|---|---|---|---|---|---|---|---|---|
| | | | | 3–20 µm filter | 0.8–3 µm filter | MAG-AL1 | MAG-AL2 | MAG-AL1 | MAG-AL2 | DO (%) | Salinity (‰) | Lake temperature (°C) |
| Surface | 0 m | Jan 2015 | 47,587,098 | 1 | 4 | 47 (49) | 46 (45) | 76 | 1 | NM | 10 | 2 |
| Oxic1: oxic zone below the surface | 5 m | Nov 2008 | 127,235,128 | 1 | 5 | 175 | 174 (178) | 99 | 1 | 98 | 22 | −0.4 |
| | 5 m | Jul 2014 | 61,203,652 | 1 | 4 | 74 (76) | 73 (71) | 87 | 5 | NM | 15 | NM |
| | 5 m | Aug 2014 | 48,117,430 | 2 | 5 | 77 (80) | 76 (74) | 80 | 4 | 55 | 19 | 2 |
| | 5 m | Oct 2014 | 48,164,156 | 12 | 51 | 699 (696) | 694 (650) | 98 | 2 | 51 | 19 | 0.9 |
| | 5 m | Dec 2014 | 47,327,506 | 9 | 16 | 260 (270) | 256 (250) | 98 | 2 | 48 | 21 | 3 |
| Oxic2: oxic zone just above the oxic-anoxic interface | 11.8 m | Nov 2008 | 137,274,340 | 2 | 8 | 325 (326) | 324 (368) | 92 | 4 | 93 | 22 | −0.3 |
| | 12.5 m | Nov 2013 | 58,343,156 | 5 | 9 | 180 (193) | 181 (347) | 74 | 24 | 52 | 23 | 1 |
| | 12.5 m | Jul 2014 | 47,781,682 | 3 | 6 | 102 (105) | 102 (103) | 93 | 6 | NM | 21 | NM |
| | 13 m | Aug 2014 | 52,404,196 | 12 | 16 | 339 (349) | 340 (472) | 82 | 16 | 43 | 24 | 4 |
| | 12 m | Oct 2014 | 44,347,552 | 11 | 32 | 402 (419) | 400 (397) | 90 | 9 | 46 | 21 | 2 |
| | 12 m | Dec 2014 | 47,658,964 | 19 | 44 | 659 (665) | 655 (637) | 91 | 8 | 48 | 22 | 2 |
| Interface: oxic-anoxic interface | 12.8 m | Nov 2008 | 103,425,100 | 1 | 0.3 | 24 (25) | 24 (31) | 68 | 29 | 21 | 26 | 3 |
| | 13.5 m | Nov 2013 | 57,773,442 | 1 | 1 | 31 (32) | 31 (46) | 60 | 37 | 42 | 30 | 3 |
| | 13.5 m | Jul 2014 | 46,322,644 | 3 | 5 | 79 (80) | 79 (92) | 74 | 24 | NM | 29 | NM |
| | 14.5 m | Aug 2014 | 57,694,754 | 2 | 6 | 106 | 106 (123) | 68 | 30 | 44 | 27 | 4 |
| | 13 m | Oct 2014 | 49,439,564 | 11 | 25 | 382 (402) | 383 (534) | 75 | 24 | 46 | 24 | 4 |
| | 13.4 m | Dec 2014 | 114,674,108 | 1 | 1 | 57 | 58 (64) | 51 | 49 | 46 | 29 | 5 |
| Anoxic1: anoxic | 14.1 m | Nov 2008 | 120,535,586 | 2 | 2 | 86 | 86 (87) | 80 | 18 | 7 | 31 | 3 |
| | 15 m | Nov 2013 | 46,777,342 | 4 | 4 | 66 | 67 (72) | 64 | 33 | 9 | 33 | 4 |

| | | | | | | | | | | | | |
|---|---|---|---|---|---|---|---|---|---|---|---|---|
| zone just below the oxic-anoxic interface | 16 m | Oct 2014 | 42,004,690 | 3 | 3 | 48 | 48 (51) | 78 | 22 | 5 | 27 | 4 |
| | 14 m | Dec 2014 | 44,499,368 | 3 | 5 | 72 | 72 (82) | 49 | 48 | 42 | 31 | 5 |
| Anoxic2: anoxic zone | 18 m | Nov 2008 | 136,427,020 | 3 | 4 | 249 (245) | 249 (244) | 85 | 14 | 7 | 34 | 3 |
| | 19 m | Nov 2013 | 61,533,520 | 3 | 3 | 57 | 57 (60) | 70 | 26 | 0 | 36 | 3 |
| | 19 m | Oct 2014 | 46,024,210 | 4 | 2 | 43 (44) | 44 (47) | 71 | 24 | 3 | 25 | 3 |
| | 19 m | Dec 2014 | 53,825,658 | 4 | 6 | 95 | 95 (96) | 75 | 22 | 1 | 35 | 3 |
| Anoxic3: lake bottom-most depth | 23 m | Nov 2008 | 119,158,216 | 1 | 2 | 64 (65) | 64 (69) | 76 | 19 | 11 | 40 | 3 |
| | 24 m | Nov 2013 | 43,627,018 | 8 | 3 | 42 | 42 (44) | 83 | 15 | 0 | 42 | 2 |
| | 24 m | Oct 2014 | 51,586,990 | 1 | 2 | 24 | 24 (27) | 79 | 18 | 1 | 34 | 2 |
| | 24 m | Dec 2014 | 60,254,882 | 2 | 4 | 52 | 53 (55) | 86 | 13 | 0 | 40 | 2 |

[A] Total number of reads in merged metagenomes. The metagenomes from 3–20 and 0.8–3 μm filter fractions from a time period and depth were combined to form merged metagenomes. Only metagenomes with ≥ 1% *Synechococcus*-like OTU abundance were selected. [B] *Synechococcus*-like OTU relative abundance values were taken from previously published data [3]. *Synechococcus*-like OTU abundance was very low (≤ 0.3%) in 0.1–0.8 μm filter fraction metagenomes (not shown here) from all time periods and depths. [C] Median and mean read depths of MAG-AL1 and MAG-AL2 were calculated in each merged metagenome. A single value is shown for cases where the mean and median values were same. Due to high ANI between MAG-AL1 and MAG-AL2 (Additional file 1: Figs. S1, S4) the median and mean read depths represent the overall *Ca*. Regnicoccus frigidus population in Ace Lake merged metagenomes. Therefore, SNP frequencies were used to calculate the contribution and read depths of *Ca*. Regnicoccus frigidus phylotypes. [D] The values indicate the contribution of *Ca*. Regnicoccus frigidus phylotypes to the total *Ca*. Regnicoccus frigidus population in a merged metagenome. The percentages are the minimum of SNP frequencies at positions 217 and 231 of 16S rRNA genes of MAG-AL1 and MAG-AL2 (Additional file 1: Table S3; see "Methods" for further description). MAG-AL2 values with light red background indicate that the read depth of MAG-AL2 was < 20 in these merged metagenomes. [E] Ace Lake physicochemical data, i.e., dissolved oxygen (DO), salinity and lake temperature [3] were gathered from each lake depth and time period during sample collection. The DO values measured using a YSI Sonde in 2008 and a TOA WQC in 2013–2015 were normalised before analysis (see "Methods" for further description). NM, not measured.

**Table S5** Potential *Ca*. Regnicoccus frigidus contigs from Ace Lake metagenomes identified through GC-read depth analysis.

| Sample collection date (DD/MM/YYYY); depth; filter fraction[A] | MAG completeness[B] | Number of potential *Ca*. Regnicoccus frigidus contigs[C] | Total length of potential *Ca*. Regnicoccus frigidus contigs (bp)[D] |
|---|---|---|---|
| 19/11/2008; 5 m; 3 µm | 96 | 9 | 265,268 |
| 19/11/2008; 5 m; 0.8 µm | 98 | 2 | 38,416 |
| 21/11/2008; 11.8 m; 3 µm | 73 | 17 | 753,414 |
| 21/11/2008; 11.8 m; 0.8 µm | 98 | 13 | 231,641 |
| 21/11/2008; 11.8 m; 0.1 µm | - | 8 | 109,408 |
| 21/11/2008; 12.8 m; 3 µm | 97 | 2 | 47,989 |
| 21/11/2008; 12.8 m; 0.8 µm | 84 | 2 | 23,525 |
| 21/11/2008; 14.1 m; 3 µm | 75 | 13 | 635,296 |
| 21/11/2008; 14.1 m; 0.8 µm | 99 | 3 | 43,023 |
| 21/11/2008; 14.1 m; 0.1 µm | - | 1 | 16,798 |
| 21/11/2008; 18 m; 3 µm | 93 | 23 | 620,333 |
| 21/11/2008; 18 m; 0.8 µm | 98 | 6 | 85,544 |
| 23/11/2008; 23 m; 3 µm | 99 | 3 | 46,076 |
| 23/11/2008; 23 m; 0.8 µm | 99 | 5 | 81,968 |
| 25/11/2013; 12.5 m; 3 µm | 99 | 4 | 69,327 |
| 25/11/2013; 12.5 m; 0.8 µm | 96 | 8 | 162,922 |
| 26/11/2013; 13.5 m; 0.8 µm | 95 | 2 | 40,715 |
| 26/11/2013; 15 m; 3 µm | 98 | 1 | 20,831 |
| 26/11/2013; 19 m; 3 µm | 98 | 1 | 11,090 |
| 26/11/2013; 19 m; 0.8 µm | 99 | 1 | 24,231 |
| 2/07/2014; 5 m; 3 µm* | 99.7 | 1 | 20,832 |
| 2/07/2014; 5 m; 0.8 µm | 99.6 | 3 | 47,071 |
| 3/07/2014; 12.5 m; 3 µm | 99.7 | 2 | 36,549 |
| 3/07/2014; 13.5 m; 3 µm | 97 | 1 | 20,832 |
| 20/08/2014; 5 m; 3 µm | 98 | 1 | 20,850 |
| 20/08/2014; 5 m; 0.8 µm | 99.7 | 1 | 18,177 |
| 21/08/2014; 13 m; 3 µm | 99 | 1 | 10,469 |
| 20/10/2014; 12 m; 0.8 µm | 99 | 1 | 11,465 |
| 21/10/2014; 13 m; 0.8 µm | 98 | 8 | 123,045 |
| 21/10/2014; 13 m; 0.1 µm | - | 1 | 22,223 |
| 21/10/2014; 16 m; 3 µm | 96 | 1 | 20,850 |
| 21/10/2014; 16 m; 0.8 µm | 99 | 1 | 10,429 |
| 21/10/2014; 19 m; 3 µm | 98 | 2 | 32,101 |
| 21/10/2014; 19 m; 0.8 µm | 95 | 1 | 10,307 |
| 21/10/2014; 19 m; 0.1 µm | - | 1 | 18,359 |
| 21/10/2014; 24 m; 3 µm | - | 14 | 188,830 |
| 21/10/2014; 24 m; 0.8 µm | 98 | 1 | 10,583 |
| 4/12/2014; 5 m; 0.8 µm | 99.7 | 1 | 10,143 |
| 4/12/2014; 5 m; 0.1 µm | - | 1 | 23,945 |
| 4/12/2014; 12 m; 0.8 µm | 99.7 | 1 | 11,465 |
| 4/12/2014; 13.4 m; 3 µm | 94 | 1 | 20,850 |
| 4/12/2014; 13.4 m; 0.8 µm | - | 2 | 22,359 |
| 3/12/2014; 19 m; 0.8 µm | 99 | 1 | 11,440 |
| 3/12/2014; 24 m; 3 µm | 88 | 5 | 106,389 |

| | | | |
|---|---|---|---|
| 27/01/2015; 0 m; 3 µm | - | 1 | 32,416 |
| 27/01/2015; 0 m; 0.8 µm | 99 | 1 | 20,850 |

[A] GC-read depth analysis was performed using contigs from individual metagenomes. * MAG-AL1 was generated from this metagenome. [B] *Ca*. Regnicoccus frigidus MAGs were assembled one MAG per metagenome, except for some metagenomes from which high- or medium-quality *Ca*. Regnicoccus frigidus MAGs were not assembled (indicated by '-' symbol in the column). [C] Potential *Ca*. Regnicoccus frigidus contigs refer to cyanobacterial contigs that had ≥ 96% identity matches (across > 50% of contig length) to *Ca*. Regnicoccus frigidus MAGs. [D] Sum of lengths of potential *Ca*. Regnicoccus frigidus contigs identified in each metagenome through GC-read depth analysis. For comparison, the average length of *Ca*. Regnicoccus frigidus MAGs with ~100% bin completeness is 2.8 Mb.

**Table S6** Description of *Ca.* Regnicoccus frigidus metabolic capacity and metadata.

| | |
|---|---|
| Species name | *Candidatus* Regnicoccus frigidus |
| Species etymology | fri'gi.dus. L. masc. adj. frigidum cold; referring to the cold environment |
| Species status | sp. nov. |
| Genome type | Metagenome-assembled genome |
| Genome status | Draft |
| GenBank accession ID | JAOANE000000000 |
| IMG bin ID | 3300023237_10 |
| Bin completeness | 99.73% |
| Bin contamination | 0.09% |
| Total base pair count | 2,644,322 bp |
| Number of contigs and genes | 64 contigs; 2,929 genes |
| GC mol % | 63.79% |
| Region of origin | Antarctica |
| Geographic location | Ace Lake |
| Latitude | 68°28′ S |
| Longitude | 78°11′ E |
| Habitat | Meromictic, saline lake |
| Sampling date | 2 July 2014 |
| Lake Depth | 5 m |
| Lake temperature | Not measured during sampling due to logistic issues [3] |
| Metabolic capacity | Aerobic oxygenic photoautotroph (using Calvin-Benson-Bassham cycle) in the light, chlorophyll-based;<br>Possible aerobic heterotroph under dark conditions, using exogenous sugars and glycerol, which might also be used as precursors for compatible solute biosynthesis (e.g., glucosylglycerol);<br>Possible facultative anaerobe under dark and anoxic conditions: fermentation using stored glycogen coupled to evolution of $H_2$;<br>Glycolysis via Entner-Doudoroff pathway;<br>Pentose phosphate pathway;<br>Tricarboxylic acid cycle (oxidative);<br>Aerobic respiration;<br>C sources: $CO_2$, urea, cyanate, sugars, glycerol;<br>Glycogen storage;<br>N sources: nitrate, ammonia, urea, cyanate, amino acids, peptides, free cyanide, nitriles;<br>S sources: sulfate (by assimilatory sulfate reduction), arylsulfates (by arylsulfatase and assimilatory sulfate reduction);<br>Sulfide oxidation to sulfur/polysulfide (possibly for detoxification);<br>ABC transporters (urea, amino acids, sugars);<br>Other transporters (peptides, glycerol, nitrate/nitrite, ammonium);<br>BREX type 1 system;<br>Retron anti-phage system;<br>Type I restriction-modification system. |
| Sequencing technology | Illumina HiSeq 2500-1TB |
| Assembly software used | BFC version r181 [5]; SPAdes v3.11.1 [6, 7] |
| Binning software used | MetaBAT v0.32.5 [8]; CheckM v1.0.11 [9] |

The *Ca.* Regnicoccus frigidus data are presented as per the recommendations for describing novel *Candidatus* species [10].

**References:**


1. Tang J, Du LM, Liang YM, Daroch M. Complete genome sequence and comparative analysis of *Synechococcus* sp. CS-601 (SynAce01), a cold-adapted cyanobacterium from an oligotrophic Antarctic habitat. Int J Mol Sci. 2019;20:152.
2. Powell LM, Bowman JP, Skerratt JH, Franzamnn PD, Burton HR. Ecology of a novel *Synechococcus* clade occurring in dense populations in saline Antarctic lakes. Mar Ecol Prog Ser. 2005;291:65–80.
3. Panwar P, Allen MA, Williams TJ, Hancock AM, Brazendale S, Bevington J, et al. Influence of the polar light cycle on seasonal dynamics of an Antarctic lake microbial community. Microbiome. 2020;8:116.
4. Goldfarb T, Sberro H, Weinstock E, Cohen O, Doron S, Charpak-Amikam Y, et al. BREX is a novel phage resistance system widespread in microbial genomes. EMBO J. 2015;34:169–83.
5. Li H. BFC: correcting Illumina sequencing errors. Bioinform. 2015;31:2885–7.
6. Nurk S, Bankevich A, Antipov D, Gurevich AA, Korobeynikov A, Lapidus A, et al. Assembling single-cell genomes and mini-metagenomes from chimeric MDA products. J Comput Biol. 2013;20:714–37.
7. Nurk S, Meleshko D, Korobeynikov A, Pevzner PA. MetaSPAdes: a new versatile metagenomic assembler. Genome Res. 2017;27:824–34.
8. Kang DD, Li F, Kirton E, Thomas A, Egan R, An H, et al. MetaBAT 2: an adaptive binning algorithm for robust and efficient genome reconstruction from metagenome assemblies. PeerJ. 2019;7:e7359.
9. Parks DH, Imelfort M, Skennerton CT, Hugenholtz P, Tyson GW. CheckM: assessing the quality of microbial genomes recovered from isolates, single cells, and metagenomes. Genome Res. 2015;25:1043–55.
10. Chuvochina M, Rinke C, Parks DH, Rappé MS, Tyson GW, Yilmaz P, et al. The importance of designating type material for uncultured taxa. Syst Appl Microbiol. 2019;42:15–21.
11. Salazar VW, Tschoeke DA, Swings J, Cosenza CA, Mattoso M, Thompson CC, et al. A new genomic taxonomy system for the *Synechococcus* collective. Environ Microbiol. 2020;22:4557–70.
12. Panwar P, Allen MA, Williams TJ, Haque S, Brazendale S, Hancock AM, et al. Remarkably coherent population structure for a dominant Antarctic *Chlorobium* species. Microbiome. 2021;9:231.
13. Roux S, Páez-Espino D, Chen IMA, Palaniappan K, Ratner A, Chu K, et al. IMG/VR v3: an integrated ecological and evolutionary framework for interrogating genomes of uncultivated viruses. Nucleic Acids Res. 2021;49:D764–75.
14. Páez-Espino D, Eloe-Fadrosh EA, Pavlopoulos GA, Thomas AD, Huntemann M, Mikhailova N, et al. Uncovering Earth's virome. Nature. 2016;536:425–30.
